# JAK-STAT signaling in inflammation and stress-related diseases: implications for therapeutic interventions

**DOI:** 10.1186/s43556-023-00151-1

**Published:** 2023-11-08

**Authors:** Alexey Sarapultsev, Evgenii Gusev, Maria Komelkova, Irina Utepova, Shanshan Luo, Desheng Hu

**Affiliations:** 1 Russian-Chinese Education and Research Center of System Pathology, South Ural State University, 454080 Chelyabinsk, Russia; 2 Institute of Immunology and Physiology, Ural Branch of the Russian Academy of Science, 620049 Ekaterinburg, Russia; 3 Department of Organic and Biomolecular Chemistry, Ural Federal University, 620002 Ekaterinburg, Russian Federation; 4 Institute of Hematology, Union Hospital, Tongji Medical College, Huazhong University of Science and Technology, Wuhan, 430022 China; 5 Department of Integrated Traditional Chinese and Western Medicine, Union Hospital, Tongji Medical College, Huazhong University of Science and Technology, Wuhan, 430022 China; 6 Key Laboratory of Biological Targeted Therapy, The Ministry of Education, Wuhan, 430022 China; 7 Clinical Research Center of Cancer Immunotherapy, Hubei Wuhan, 430022 China

**Keywords:** Inflammation, JAK inhibitors, JAK-STAT signaling, Neuropsychiatric disorders, Stress-related conditions, Therapeutic advancements

## Abstract

The Janus kinase-signal transducer and transcription activator pathway (JAK-STAT) serves as a cornerstone in cellular signaling, regulating physiological and pathological processes such as inflammation and stress. Dysregulation in this pathway can lead to severe immunodeficiencies and malignancies, and its role extends to neurotransduction and pro-inflammatory signaling mechanisms. Although JAK inhibitors (Jakinibs) have successfully treated immunological and inflammatory disorders, their application has generally been limited to diseases with similar pathogenic features. Despite the modest expression of JAK-STAT in the CNS, it is crucial for functions in the cortex, hippocampus, and cerebellum, making it relevant in conditions like Parkinson's disease and other neuroinflammatory disorders. Furthermore, the influence of the pathway on serotonin receptors and phospholipase C has implications for stress and mood disorders. This review expands the understanding of JAK-STAT, moving beyond traditional immunological contexts to explore its role in stress-related disorders and CNS function. Recent findings, such as the effectiveness of Jakinibs in chronic conditions such as rheumatoid arthritis, expand their therapeutic applicability. Advances in isoform-specific inhibitors, including filgotinib and upadacitinib, promise greater specificity with fewer off-target effects. Combination therapies, involving Jakinibs and monoclonal antibodies, aiming to enhance therapeutic specificity and efficacy also give great hope. Overall, this review bridges the gap between basic science and clinical application, elucidating the complex influence of the JAK-STAT pathway on human health and guiding future interventions.

**Graphical Abstract:**

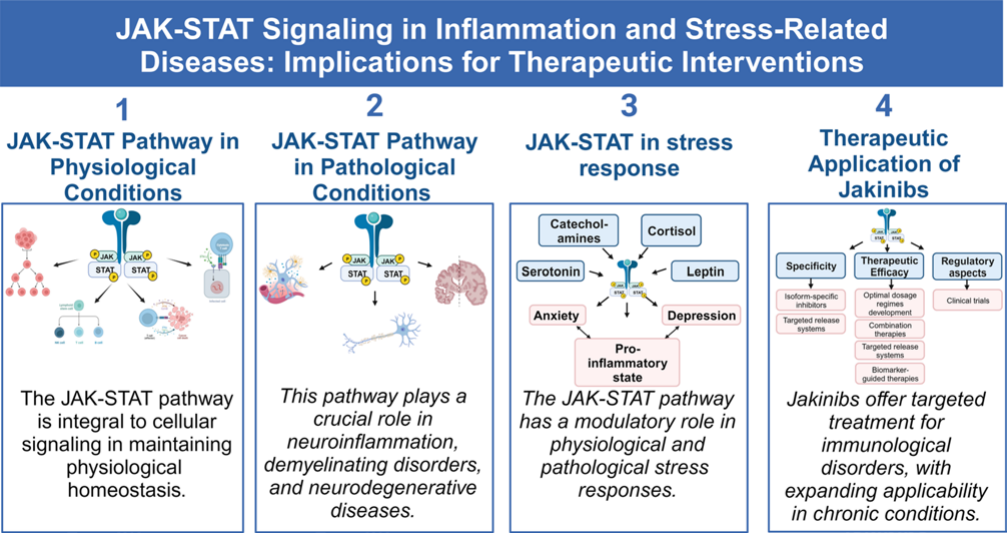

**Supplementary Information:**

The online version contains supplementary material available at 10.1186/s43556-023-00151-1.

## Introduction

The Janus kinase-signal transducer and transcription activator pathway (JAK-STAT) is a seminal axis in cellular signal transduction, facilitating the transmission of signals from membrane-bound receptors to the nucleus and governing cellular activities such as proliferation, differentiation, and apoptosis [1]. Comprising four members, JAK1, JAK2, JAK3 and TYK2, the JAK family of nonreceptor tyrosine kinases (nRTKs) is differentially expressed across various cell types and plays distinct roles in cytokine signaling and cellular processes. These include immune modulation, antiviral responses, and hematopoiesis, among others [1, 2]. Although the pathway is fundamental to both innate and adaptive immune responses, mutations can lead to detrimental outcomes, such as severe immunodeficiencies and oncogenic transformations [1–7].

The existing literature on JAK-STAT signaling is rich in insights into its role in infectious diseases, autoimmune disorders, and cancers. However, the implications of the pathway for stress and stress-related disorders remain underexplored [3, 4]. Therefore, this review aims to fill this research gap by focusing on the role of the JAK-STAT pathway in inflammation and stress-related conditions, emphasizing the shared mediators and mechanisms between stress and inflammation, as well as the attributes of depression-like states associated with inflammation [5–7].

Our overarching objective is to provide a comprehensive and up-to-date analysis that serves as a stepping stone for future research and therapeutic advancements. Specifically, this review will advocate for expanding the scope of research on the involvement of the JAK-STAT pathway in stress-related conditions, thereby unearthing new avenues for the application of JAK inhibitors (Jakinibs) across a broader array of diseases.

## Psychoemotional stress, its connection to cellular pro-inflammatory stress, and neuroinflammation

### Physiological and pathological significance of mental stress

Stress-related disorders encompass a variety of conditions, including posttraumatic stress disorder, acute stress reaction, adjustment disorder, and depression. These disorders commonly manifest themselves after traumatic or stressful life events [8]. Chronic psychological stress and depression are notably associated with increased pro-inflammatory states within specific regions of the brain [9]. Furthermore, these disorders are associated with functional anomalies on the hypothalamic–pituitary–adrenal (HPA) axis and elevated sympathetic nervous system activity, both of which contribute to the development and exacerbation of mental and psychosomatic illnesses [10].

It is worth noting that stress may possess dual characteristics: adaptive and maladaptive (distress). The distinction between these forms hinges on several parameters, such as duration and severity [11]. Identifying the nuances between the adaptive and maladaptive aspects of mental stress remains a challenging task [12]. Clinical manifestations of distress include depression and other mental disorders related to stress.

### Pro-inflammatory cell-tissue stress and inflammation

Recent advances in molecular biology and pharmacology have provided a comprehensive framework to understand cellular stress and inflammation [13]. The conservative mechanisms underlying pro-inflammatory cellular stress serve as a common response across various types of cells to real or potential damage. This response is characterized by oxidative stress, the activation of inducible intracellular signaling pathways, and the establishment of a pro-inflammatory cellular phenotype, both secretory and receptor-based. These mechanisms facilitate cellular survival and tissue adaptation under challenging conditions, but may also result in various outcomes such as programmed apoptosis, necrosis, cellular senescence, transdifferentiation, and malignancy. At the tissue level, these processes can manifest themselves as tumor growth, tissue sclerosis, tissue atrophy, and different variants of inflammatory processes [14].

Inflammation can be broadly classified into at least three primary categories [14]. Canonical inflammation is delineated by focal exudative-vascular reactions and marked leukocyte migration to the site of tissue injury. Life-threatening systemic microvascular inflammation is characterized by shockogenic conditions, intravascular coagulation, and cytokine storms [15, 16]. In contrast, low-grade chronic inflammation arises from metabolic stressors and other low-intensity stressors and lacks a barrier function in the focus of inflammation. It exhibits transitional states ranging from physiological conditions to the onset of canonical inflammation [17]. Of particular interest, low-grade chronic inflammation is implicated not only in classical neurodegenerative disorders but also in latent neurodegeneration associated with stress-induced depressive states [18].

### The role of pro-inflammatory cellular stress in ensuring homeostasis of nervous tissue and pathology

Despite the protective measures inherent to neural tissue, such as the blood–brain barrier and unique metabolic properties that minimize aerobic breakdown of fatty acids, as well as the comparatively low pro-inflammatory activity of stromal macrophages (microglia), tissue remains highly vulnerable to a variety of damaging factors and alterations in multiple homeostatic parameters. This susceptibility is partly attributed to the elevated metabolic demands of the tissue, specifically high rates of energy, glucose, and oxygen consumption. Additionally, the tissue experiences rapid protein metabolism and fluctuating ion concentrations, including calcium within neurons [19–21].

Given this context, it is not surprising that imbalances in neurotransmitter activity can precipitate excitotoxicity and localized neural tissue damage, conditions that are frequently observed in stress and depression [22, 23]. In particular, neurotransmitter signaling pathways often incorporate protein kinases, transcription factors, and other regulatory elements, thereby facilitating the development of cellular pro-inflammatory stress. This is especially pertinent for neurotransmitters that operate through metabotropic receptors linked to G proteins, which are involved in neurotransduction and in the maintenance of neural cell homeostasis [24].

In light of these observations, a pro-inflammatory secretory and receptor phenotype is also manifested in neural cells, including neurons. Unlike other cell types, these neural cells can contribute to the cytokine network under specific conditions such as neurogenic stress, pain, migraines, and both neurodegenerative and psychiatric disorders [25–28]. Extended periods of psychoemotional stress, particularly in regions of the brain such as the hippocampus and other components of the limbic system, induce the production of pro-inflammatory cytokines. These cytokines are believed to play a role in the pathogenesis of trauma-induced anxiety and the onset of depression, in part through negative feedback mechanisms [29].

### Interrelationship between mental stress, depression, and pro-inflammatory cellular tissue stress

The relationship between mental stress, depression, and pro-inflammatory stress of cellular tissues can be intricately complex, as these biological phenomena often intersect and influence each other. In the realm of mental health, persistent and dysregulated mental stress can lead to functional impairments and structural alterations in particular regions of the brain. These ramifications are not isolated events; rather, they engage pro-inflammatory tissue stress mechanisms, providing a substrate for different forms of neuroinflammation. It should be noted that the onset of such neuroinflammatory states often involves multilateral physiological disruptions, such as dysregulation of the hypothalamic–pituitary–adrenal (HPA) axis, compromised integrity of the intestinal barrier, altered hemodynamics, and other related processes.

In addition, a pathogenetic link is established between distress in the neuroendocrine system and localized and systemic inflammatory manifestations in the extracerebral organs. These interconnected phenomena underscore the complexity of the relationships between mental stress, depression, and various pathological states, including inflammation, tissue senescence, and allostasis. By understanding these intricate connections, researchers can better target mechanisms for therapeutic intervention, particularly in conditions characterized by chronic mental stress and its consequent systemic manifestations.

### Possible role of the JAK -STAT pathway as pro-inflammatory factors in maintaining homeostasis in nervous tissue and in the pathogenesis of stress-associated neuropsychiatric diseases

The JAK-STAT pathway occupies a pivotal position at the convergence of cytokine-mediated signaling and neurotransduction, facilitating the intricate regulation of neuroinflammation and its associations with stress-induced neuropsychiatric conditions. This comprehensive exploration amalgamates the evolutionary perspective of the pathway with its diverse roles in maintaining homeostasis within nervous tissues and its involvement in stress-related neuropsychiatric disorders. From an evolutionary point of view, the JAK-STAT pathway predates the divergence of protostomes and deuterostomes, underscoring its involvement not only in canonical inflammation but also in primordial forms of immunity [30, 31]. Its evolutionary trajectory aligns with the emergence of vertebrates, characterized by a diversification of pathway components along with a proliferation of cytokines and their corresponding receptors [32]. In addition, the ancestral origins of JAK-STAT interactions with the glutamate receptor system, the NMDA receptor (NMDAR) and other pathways related to oxidative stress and hypoxia are discernible, even in phylogenetically distant species such as zebrafish [33].

Going beyond traditional inflammation, the JAK-STAT pathway shows remarkable functional versatility. In oncological contexts, IL-6/JAK-STAT3 signaling plays a key role in the regulation of various aspects of tumor biology, including cell proliferation, metastasis, and the concurrent suppression of antitumor immune responses [34]. Furthermore, its influence extends into embryogenesis, particularly in its modulation of the highly conserved Notch signaling pathway [35]. Critically, the JAK-STAT pathway is indispensable for maintaining homeostasis in tissue, its aberrant activation being implicated in a wide range of pathologies including obesity, diabetes, and low-grade systemic chronic inflammation, which may manifest in various tissues, including the nervous system [36, 37]. Furthermore, the pathway plays a role in the instigation of low-grade systemic chronic inflammation, a condition associated with sarcopenia, and also participates in the complex pathogenetic relationship between depression and systemic manifestations of chronic inflammation [38–41].

In the context of maintaining homeostasis in nervous tissues, the JAK-STAT pathway, activated primarily in glial cells such as astrocytes and microglia, assumes a pivotal role in regulating neuroinflammation [42–46]. The pathway orchestrates a delicate balance between pro-inflammatory and anti-inflammatory cytokines in response to various physiological signals, shaping the neuroinflammatory environment within nervous tissues [46]. In particular, the JAK-STAT pathway serves as a regulatory hub for transduction signals, affecting the activation of various inflammatory factors, growth factors, and angiogenic factors in the tumor microenvironment. It also participates in the regulation of the maturation, proliferation, and differentiation of various immune cells [43, 44]. In the realm of chronic stress and neuropsychiatric diseases, dysregulation of the pathway becomes a critical factor in the pathogenesis [45]. Chronic stress leads to sustained activation of the pathway in astrocytes and microglia, perpetuating neuroinflammation, which in turn contributes to neuronal dysfunction, synaptic disorders, and cognitive impairments, hallmark features of stress-associated neuropsychiatric disorders. Concurrently, the pathway's pro-inflammatory actions extend to the modulation of synaptic plasticity in neurons, with stress-induced activation having the potential to disrupt the balance of synaptic proteins and thus contribute to the pathogenesis of neuropsychiatric diseases [46].

In conclusion, these considerations underscore the need for a nuanced exploration of the JAK-STAT signaling pathway, particularly its multifaceted implications within the realms of neuroinflammation and stress-related neuropsychiatric disorders.

### Jak kinase structure and regulatory mechanisms

#### Jak gene locations and protein structure

The four genes of the Jak family in mammals are distributed on three different chromosomes. Tyk2, the first member of the Jak family identified as a new class of nRTK in humans, is located on chromosome 19p13.2, clustered with the Jak3 gene at 19p13.1 [47]. The genes encoding Jak1 and Jak2 are found on chromosomes 1p31.3 and 9p24, respectively [48].

Seven distinct Jak homology regions (JH) are structural domains within members of the Jak family, playing a vital role in their functioning and determining their unique functionalities and interactions within signaling pathways [35, 49–58]. The description of the JAK homology region, along with the foundational structure of the STAT domains, is as follows:

- JH1 (Tyrosine Kinase Domain): JH1, extensively studied, has tyrosine kinase activity, phosphorylating Jak and downstream molecules. It regulates activation and signaling pathways. The JH1 domain contains structural components and conserved residues for kinase activity, including the ATP-binding site and the catalytic loop, critical for phosphorylation. It also has regulatory elements that modulate Jak signaling through autophosphorylation and interactions.
- JH2 (Pseudokinase Domain): Catalytically inactive JH2 negatively regulates Jak activity [50]. It interacts with Jak and regulatory proteins, which impacts Jak activity. Mutations in JH2 lead to hyperactive JAK-STAT pathways [50].
- JH3 (Ferm-adjacent Region): Adjacent to FERM, JH3 stabilizes Jak and mediates protein interactions [51, 52]. It contributes to Jak's association with cellular compartments, crucial for proper function [52].
- JH4 (Src Homology 2-like Domain): JH4 resembles SH2, aiding Jak in binding phosphorylated tyrosine residues to activated receptors, initiating signaling.
- JH5 (SH2-like Domain): Like SH2, JH5 mediates protein interactions and recruits downstream molecules to activated Jak [35, 49, 52–55]. It regulates Jak kinase activity and autoinhibition.
- JH6 (Unique Region): The role of JH6 is under study; it likely regulates interactions within signaling pathways [56], affecting Jak stability and conformation upon binding of the ligand.
- JH7 (Transactivation Domain): JH7 interacts with transcription factors, transactivating downstream genes [57], influencing cellular processes [58].

These seven Jak homology regions collectively contribute to the structural organization, activity, regulation, and interaction capabilities of JAK proteins, which play an indispensable role in cellular signaling processes.

#### Jak regulatory mechanisms

**Fig. 1:**
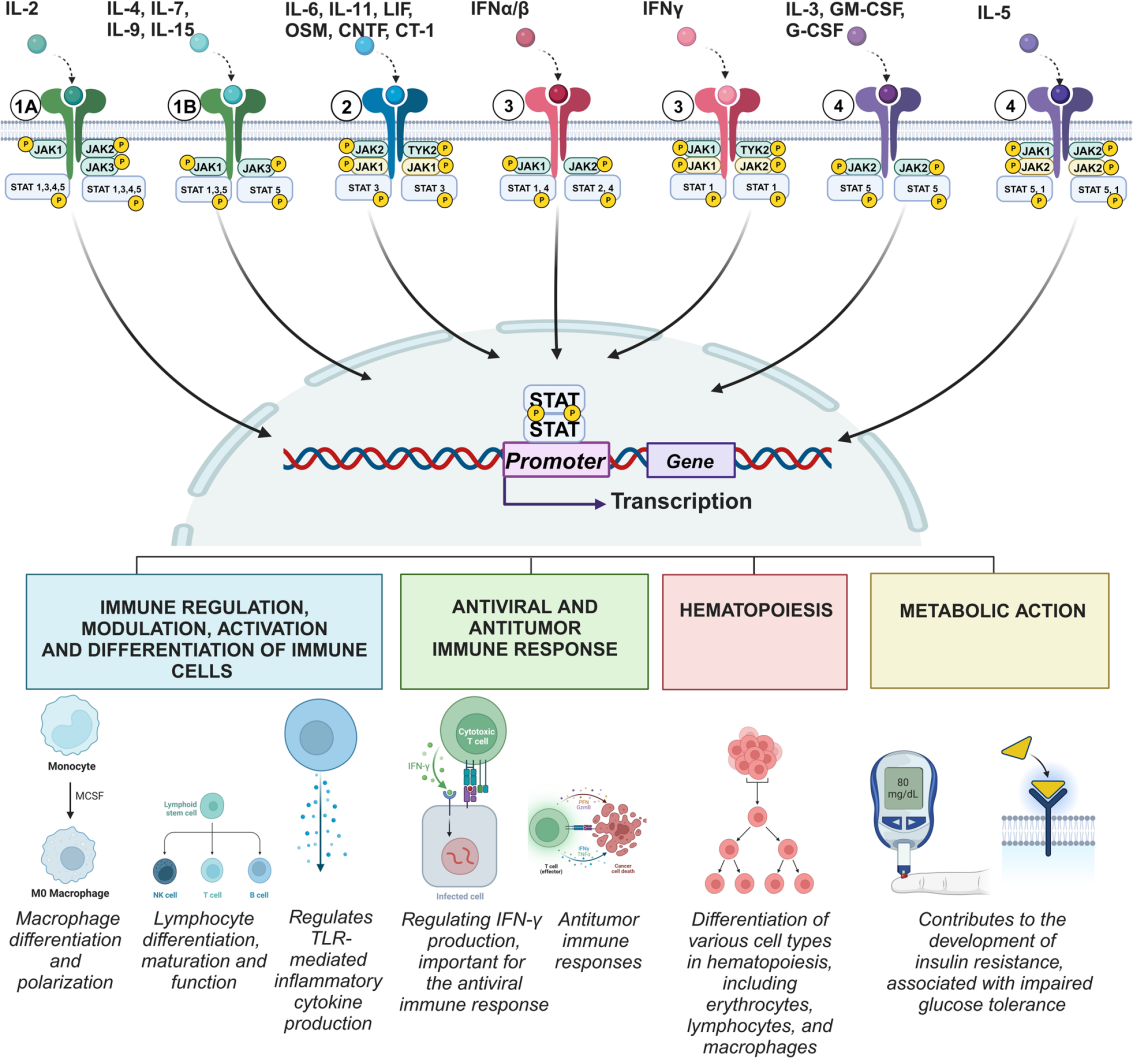
Cytokine signaling through the JAK-STAT pathway in physiological conditions. Note: Top: 1A—receptors IL-2 (IL-2Rαβγ); 1B—receptors γc -family cytokines; 2–gp130 subunit receptors; 3—type II cytokine receptors; 4—type I cytokine receptors. Under standard physiological conditions, the JAK-STAT pathway acts as an indispensable cellular communication mechanism. Highlighted are distinct regulatory combinations integral to its functionality, including JAK1-JAK2-JAK3/IL-2, IL-4, IL-7, IL-9, IL-15/JAK1, and JAK3. Center: Central to the depiction is the sophisticated intracellular machinery responsible for transcriptional and translational cascades, which are activated by specific cytokines. Bottom: Four cardinal roles of the JAK-STAT pathway under normal physiological conditions, undisturbed by external stressors, are elucidated: 1) Immune regulation: Modulating, differentiating, and activating immune cells. 2) Defense mechanisms: Spearheading potent antiviral and antitumor responses that determine the body's resistance to the occurrence of infectious and tumor diseases .3) Hematopoiesis: Governing the process of blood cell formation. 4) Metabolic regulation: Navigating cellular energy dynamics. In essence, this figure underscores the JAK-STAT pathway's paramount importance in cellular physiology, particularly in conditions devoid of stress

The JAK-STAT signaling pathway is a key mediator in the transduction of numerous cytokine-mediated signals. Different cytokines can specifically activate distinct JAK and STAT proteins, leading to a diverse range of cellular responses. An overview of the associations between cytokines, JAK kinases, and STAT proteins is provided in Table 1 and illustrated in Fig. 1*.*

**Table 1:** Associations Between Cytokines, JAK Kinases, and STAT Proteins

Cytokines	JAK	STAT
IL-7, IL-9, IL-15, IL-21 (all share a γ-chain with IL-2R)	JAK1, JAK3	STAT1, STAT3 (JAK1), STAT5 (JAK1/JAK3)
IL-2 (IL-2Rαβγ)	JAK1, JAK2, JAK3	STAT1, STAT3, STAT4, STAT5
IL-3, G-CSF, GM-CSF	JAK2	STAT5 (JAK2/JAK2)
IL-4	JAK1, JAK3	STAT6
IL-5	JAK1, JAK2	STAT1 (JAK1/JAK2), STAT5 (JAK2)
IL-6, IL-11	JAK1, JAK2, TYK2	STAT3 (JAK2/TYK2; JAK1)
IL-12	JAK2, TYK2	STAT4 (JAK2/TYK2)
IL-13	JAK1, JAK2, TYK2	STAT6 (JAK1/TYK2)
IFNα/β	JAK1, TYK2	STAT1/STAT2, STAT4 (JAK2/TYK2)
IFNγ	JAK1, JAK2	STAT1/STAT1 (JAK1/JAK2)
IL-10 family: IL-20, IL-22, IL-24, IL-26	JAK1	STAT3 (JAK1)
IL-10 (IL-10R)	JAK1, TYK2	STAT1, STAT3
IL-23	JAK2, TYK2	STAT3 (JAK2/TYK2), STAT4 (JAK2/TYK2)

The parentheses indicate the specific JAKs or their combination, present on the same cytokine receptor, that activate the corresponding STAT monomer or dimer. Additional regulatory components, such as suppressor of cytokine signaling (SOCS) proteins, can also modulate JAK-STAT signaling. References: [2, 59–65]

When ligand binding, receptor dimerization activates the associated JAKs, initiating transphosphorylation that increases their kinase activity. This, in turn, enables the phosphorylation of specific tyrosine residues on the receptor, creating binding sites for proteins with Src homology 2 (SH2) domains, predominantly STAT proteins [60]. Once bound to these phosphorylated tyrosines, STATs undergo phosphorylation by activated JAKs, dissociate from the receptor, and form homodimers or heterodimers. Subsequently, they translocate to the cell nucleus to initiate gene transcription [60]. During this classical activation of JAK-STAT, there is an induction of alternative signaling pathways associated with cellular stress, notably the phosphoinositide 3-kinase/protein kinase B (PI3K/AKT) and mitogen-activated protein kinase/extracellular signal-regulated kinase pathways (MAPK/ERK) [61]. These alternative pathways share a common feature: the SH2 domain in their key regulatory proteins [61].

This intricate co-activation pattern signifies that signaling via JAK-dependent receptors not only orchestrates the classical JAK-STAT cascade, but also instigates a more complex network of trans-signaling pathways, such as PI3K/AKT and MEK/ERK [57]. The specificity of STAT activation is dependent on various factors including the composition of JAKs and receptor characteristics, as demonstrated by the differing impacts of cytokine receptors such as IL-2, IL-4, and others that use the common gamma chain (CD132) [66]. Moreover, the enzymatic activity of JAK is subject to multiple layers of intricate regulation. These include intrinsic regulatory events, such as post-translational modifications and the inhibitory function of the pseudokinase domain, as well as a large number of extrinsic regulatory elements [66]. Extrinsic Jak regulatory mechanisms include:

- SOCS-mediated Negative Feedback: SOCS proteins inhibit JAK by binding to activated JAK and cytokine receptors, limiting downstream signaling [67]. They also promote JAK degradation through the ubiquitin proteasome pathway, leading to inactivation [68]. Recent studies highlight their role in fine-tuning the immune response [69].
- Protein Phosphatases: Protein phosphatases, including PTPs and PPs, dephosphorylate tyrosine and serine/threonine residues in JAK, regulating activity [1, 70, 71]. PTP1B, SHP1, SHP2, and CD45 directly impact JAK for regulation [1, 63, 64, 71–80].
- Feedback Inhibition by Suppressors: Inhibitors containing SH2 domains target JAK phosphotyrosines for dephosphorylation, involving SH2 domain-containing protein tyrosine phosphatases (SHP) [70]. PIAS proteins suppress Jak activity by interfering with the JAK-STAT interaction or modulating JAK-STAT signaling by SUMOylation [49, 79].
- LNK (SH2B3): This adapter protein with SH2 and PH domains targets JAK phosphotyrosines [80].
- Cytokine receptor complex association: JAK is associated with cytokine receptor complexes, crucial for activation and signaling [81]. Disruptions lead to inactivation [59, 66].
- Post-translational Modifications: JAK undergoes phosphorylation, acetylation, SUMOylation, and ubiquitination to modulate activity [58].

The activation process of JAK proteins is also regulated by intramolecular interactions, which are instrumental in modulating their activity and signaling [82]. These interactions, which involve various domains within the structure of the JAK protein, play a critical role in the precision control of JAK-mediated signaling pathways and include:

- JH2-JH1 domain interaction: The pseudokinase (JH2) and kinase (JH1) domains interact within JAK to regulate function. Inactivity involves JH2 associated with JH1, inhibiting kinase activity. Ligand binding to the cytokine receptor triggers conformational changes, disrupting the JH2-JH1 interaction, and activating JAK kinases [83].
- FERM domain role: The N-terminal FERM (Four-point-one, Ezrin, Radixin, Moesin) participates in intramolecular interactions, communicating with other JAK domains, influencing JAK activity and stability [59].
- SH2 domain contribution: The JAK SH2 (Src Homology 2) domains participate in intramolecular interactions that modulate JAK activation. Inactivity entails SH2 interaction with other JAK regions, limiting the conformation. Ligand binding and receptor activation prompt conformational changes, releasing JAK activation [84].

These intrinsic interactions serve as central regulatory mechanisms that oversee JAK activation and signaling. They ensure stringent regulation of JAK-mediated pathways, thus facilitating accurate cellular responses to extracellular signals. Dysregulation of Jak regulatory mechanisms can induce aberrant signaling, which contributes to various diseases. Therefore, understanding the intricate interplay between these regulatory mechanisms and JAK proteins is essential for the creation of therapeutic strategies that target Jak signaling pathways.

### Cellular location and biological activities of JAKs

#### Localization and expression of JAKs in immune cells

JAKs reside primarily in the cytoplasm, as confirmed by research [84, 85]. Ligand binding prompts their association with cytokine receptors, which recruit them to activated receptor complexes at the plasma membrane. However, it is notable that JAKs also populate other cellular domains, such as membranes, cell surfaces, and the endoplasmic reticulum, possibly serving distinct functions in various cellular processes [83, 84]. Recent studies reveal nuclear localization of specific JAKs and their involvement in various cellular contexts. For example, nuclear expression of JAK1 occurs in large B-cell lymphoma, hinting at its nuclear tyrosine kinase role in histone phosphorylation and cell survival in human hematopoietic stem cells and B-cell leukemia cells [85]. Similarly, nuclear JAK2 localization influences histone phosphorylation and cellular survival [86]. Nuclear JAK3 is detected in cutaneous T cell lymphoma (CTCL) and primary malignant T cells in patients with Sézary syndrome, indicating its role in the pathogenesis of these conditions [87]. These findings underscore the varied subcellular placements and functions of JAKs, broadening their biological activities. Thorough exploration is needed to fully grasp the functional significance of JAK localization in cellular processes and disease scenarios.

The different members of the JAK family, JAK1, JAK2, JAK3, and TYK2, exhibit unique expression patterns in cell types. Although JAK1 and JAK2 are broadly expressed, JAK3 is confined to hematopoietic cells and lymphoid tissues [83–85]. TYK2, in contrast, is found in both immune and non-immune cells. JAK expression varies among immune cell types, reflecting their specific roles in signaling and functions (Table 2, Fig. 1) [10].

**Table 2:** Expression Patterns and Roles of JAK Family Members in Different Immune Cell Types

Immune Cell Type	JAK Expression	Role and Function	References
T cells	JAK1, JAK3 (lesser extent: JAK2, TYK2)	Crucial for T cell receptor (TCR) signaling and development	[88, 89]
		Initiate the activation of key transcription factors (STAT) essential for the differentiation of Th1 (STAT1/4), Th2 (STAT5/6), Th17 (STAT3/5), and Treg (STAT3/5) cell subsets	[17, 90]
B cells	JAK1, JAK3	Relay signals from the B-cell receptor (BCR) and cytokine receptors. JAK1 aids B cell development, JAK3 vital for maturation	[90, 91]
NK cells	JAK1, JAK3 (also: JAK2, TYK2 for NK development)	Transduce signals from cytokine receptors (IL-2, IL-15). Promote NK cell development, survival, and cytotoxicity	[92–95]
Neutrophils	JAK expression less explored	Implicated in cytokine release via JAK-STAT signaling. Influence pro-inflammatory responses	[94–97]
Monocytes	JAK1, JAK2	Essential for cytokine and growth factor signaling. Regulate monocyte activation, differentiation, and immune responses	[98–100]
Inflammatory macrophages	JAK1-3, TYK2	Initiate essential transcription factors (STAT and NF-κB) to trigger the differentiation process: M1 (STAT1, NF-κB), M2a (STAT3/6), M2b (NF-κB), and M-reg (STAT3)	[17, 90]
Dendritic cells (DC)	JAK1, JAK2, JAK3	Drive DC maturation, antigen presentation, and cytokine production	[101, 102]

#### JAK family members: diverse biological activities

JAKs play crucial roles in a wide range of biological activities by mediating signaling pathways activated by cytokines and growth factors [37, 72, 95, 103–154] (Table 3).

**Table 3:** JAKs biological activities

Biological Activity	JAK1	JAK2	JAK3	TYK2
Cytokine Signaling	Involved in signaling pathways activated by various cytokines and growth factors [74, 95, 103, 104] (Mostly type II cytokine receptors (ifnα/β, ifnγ, and IL-10), γc -family cytokines of the c family (IL-2, IL-4, IL-7, IL-9 and IL-15) and gp130-cytokines (IL-6, IL-11, LIF, OSM, CNTF, and CT-1)	Plays a critical role in the signaling of type I cytokines such as EPO, IL-3, GM-CSF; IL-5, TPO, growth hormone, prolactin, G-CSF [119, 121]	Essential for cytokine signaling, particularly the γc-cytokines (IL-2, IL-4, IL-7, IL-9, IL-15 and IL-21), IL-3, IL-5, and GM-CSF [130, 131]	Associated with various cytokine receptors including IFNAR1, IL-12Rβ1, IL-10R2, IL-23R and gp130 (IL-6, IL-11, and oncostatin M) [143, 144]
Immune response regulation	Regulates immune responses, including activation and differentiation of immune cells	Plays a role in the regulation and modulation of the immune response	Critical for immune response regulation, particularly lymphocyte maturation and function	Involved in the regulation of immune responses, including Th1/Th17 differentiation [143]
			Regulates TLR-mediated inflammatory cytokine production [132]	Is also involved in macrophage differentiation and polarization [149–151]
Antiviral Immune Response	Regulating IFN-γ production, important for the antiviral immune response [103, 108]			
Hematopoiesis	Participated in the signaling pathways that regulate hematopoietic cell development, differentiation, and proliferation. Plays a role in the regulation of hematopoietic stem cells [50, 105]	Involved in the signaling pathways that regulate adult hematopoietic stems84	Involved in early lineage decision and differentiation of various cell types in hematopoiesis, including erythrocytes, lymphocytes, and macrophages	Plays a role in myeloid cell differentiation and regulation of hematopoietic stem cells [147, 152]
	It is crucial for the differentiation, development, and function of lymphoid cells [91, 116]		It is crucial for the differentiation, development, and function of lymphoid cells (Obligate partner for Jak1)	Promotes NK cell maturation and enhances their activity [146]
	Participatory in the regulation of myeloid cell development [117, 118]	Plays a role in erythropoiesis, thrombopoiesis, and regulation of myelopoiesis [153]	Play an important role in myeloid cell differentiation [134]	
Metabolic action			Contributes to the development of insulin resistance [154]	Associated with impaired glucose tolerance [140]
Other roles	Regulating IFN-γ production, critical role in antitumor immune responses [105, 106]	Essential for erythropoietin (EPO) and thrombopoietin (TPO) receptor signaling [122–124]	Essential for γc-dependent T cells and B cells, and for γc-dependent prevention of c-dependent thymocyte apoptosis (jointly with JAK1) [89]	Involved in NK cell maturation, promotes Th1/Th17 differentiation [143–146]

JAK1 serves as a central mediator in multiple cytokine signaling pathways, including class II, γc receptor, and gp130 subunit receptors. It significantly impacts immune responses, antitumor immunity, and bone homeostasis, making it a promising candidate for selective therapeutic intervention and offering an alternative to nonselective Jakinibs to minimize side effects associated with JAK2 or JAK3 [72, 73, 94, 103, 105]. Mutations and deficiencies in JAK1 have been implicated in cancer immunity and immunodeficiency in murine models and human pathology [99–114]. Unlike JAK1, JAK2 is primarily activated by various cytokines, including erythropoietin, IL-3, and GM-CSF, and is essential for erythropoiesis, thrombopoiesis, and granulocyte differentiation. In particular, dysregulation of JAK2 leads to hematologic disorders such as myeloproliferative neoplasms and leukemia [119–129].

JAK3 specializes in associating with the common γc receptor chain and is indispensable for lymphoid functions and hematopoietic cell differentiation, modulating pro-inflammatory cytokine production in innate immune cells [121, 130, 132, 134, 137]. Furthermore, metabolic implications of the JAK-STAT signaling cascade are evident; JAK3 deficiency correlates with insulin resistance, while variations in Tyk2 and Stat6 levels are associated with metabolic disorders such as obesity and glucose intolerance [37, 139–142]. Tyk2 facilitates signals from IL-12, interferons, and other cytokines, playing a crucial role in immune cell differentiation, especially in macrophages and NK cells, and influencing early and osteogenic lineage differentiation in mouse embryonic stem cells [143–152].

In summary, members of the JAK family, including JAK1, JAK2, JAK3 and TYK2, manifest a myriad of crucial roles in various biological activities (Table 1). These roles range from cytokine signaling, immune responses, and hematopoiesis to metabolic functions, cellular growth, and survival. A comprehensive understanding of their distinct and overlapping functions is essential to delineate their contributions to both normal cellular functions and the pathogenesis of a variety of diseases.

## JAK-STAT pathway under physiological conditions in the central nervous system

The significance of the JAK-STAT signaling pathway is not restricted to the peripheral immune system but also encompasses vital functions within the central nervous system (CNS). Although initially identified for its seminal role in immune responses, this pathway has become a pivotal mediator in the CNS, governing processes such as neuroinflammation, neurogenesis, and synaptic plasticity. The dual function of the pathway, modulating both immune responses and neuronal activities, further enhances our comprehensive understanding of both physiological and pathological processes in the brain.

The expression of JAK and STAT proteins in the CNS is relatively subdued compared to other physiological systems; however, its presence is discernible in key brain regions, including the cerebral cortex, hippocampus, hypothalamus, and cerebellum. Importantly, the expression of these proteins undergoes developmental modulations: elevated levels are observed during the embryonic stages, particularly for JAK2, JAK1, STAT3, STAT6, and STAT1, which gradually decline as the organism matures [153–156]. Recent studies have revealed that JAK2 and STAT3 play an indispensable role in hippocampal synaptic plasticity, a mechanism integral to learning and memory, suggesting functions that transcend their traditional roles in gene regulation. The pathological implications of the JAK-STAT pathway in the CNS are predominantly related to neuroinflammatory processes and cell survival, extending its influence to a range of neurological disorders. These include, but are not limited to, epilepsies, brain cancer, ischemic lesions, and neurodegenerative conditions such as Alzheimer's disease.

In summary, the JAK-STAT pathway serves as a versatile signaling axis, with ramifications that range from immune modulation to complex neurobiological processes within the CNS. Its role in both physiological and pathological states provides a compelling area for future research aimed at elucidating mechanistic insights and therapeutic avenues.

### JAK-STAT in brain cells development and functioning

The development and functioning of brain cells, including neurons, astrocytes, and oligodendrocytes, are regulated by the JAK-STAT pathway. During brain development, neural stem cells (NSCs) or neural progenitor cells (NPCs) differentiate into these various cell types. In the adult brain, neurogenic regions such as the subventricular zone (SVZ) of the olfactory bulbs and the dentate gyrus (DG) of the hippocampus harbor NSC populations [156]. NSC proliferation is controlled by the JAK-STAT pathway, with cytokines such as IL-15 expressed by adult NSCs in the SVZ activating STAT1, STAT3, and STAT5. Inhibition of JAK blocks NSC proliferation [157].

JAK-STAT signaling activation plays a role in astrogliogenesis, as forced activation leads to precocious astrogliogenesis, while inhibition of the pathway blocks astrocyte differentiation. Autoregulation of the JAK-STAT pathway is suggested to control the onset of astrogliogenesis [157]. JAK1 is predominantly involved in astrocytic differentiation, while JAK2, which can be activated by the leptin receptor, is essential for neural stem cell proliferation [158–160]. Modulation of JAK3 signaling also contributes to the differentiation of neurons, oligodendrocytes, and microglial cells [161]. Furthermore, Jak3-dependent microglial activity regulates the development of neurons and neurite outgrowth [160]. In neurons, the JAK-STAT pathway is involved in the control of the release of hormones and peptides from structures of the CNS such as the hypothalamus, influencing processes such as energy homeostasis and reproduction in the CNS.

The JAK-STAT pathway is influenced by various upstream regulators, including cytokines, hormones, and growth factors. These regulators have been shown to modulate synaptic activity and alter synaptic function [161].

### The interplay between JAK-STAT and CNS Receptors

The JAK-STAT pathway plays a crucial role in regulating the inflammatory and stress response. Its interaction with receptors in both the central and peripheral nervous system contributes to its complex and versatile functionality. These receptors are distributed throughout the central and peripheral nervous system and serve as transmitters of signals essential for normal neurological function. In addition, they play a pivotal role in the detection and response of external and internal stressors. As a result, the interaction between JAK-STAT and these receptors forms a critical network that responds to inflammation and stress, influencing central and peripheral processes.

#### JAK-STAT and serotonin receptors

The interplay between serotonin and JAKs, particularly JAK2, is evident in the context of atypical antipsychotics and their effects on serotonin signaling. Serotonin receptors, specifically 5HT2A, can stimulate phospholipase C (PLC), a vital element of signal transduction pathways. Certain medications, such as atypical antipsychotic olanzapine, can desensitize the activation of PLC by the 5HT2A receptor, and this desensitization appears to be mediated, at least in part, by the JAK-STAT pathway [162–165]. This suggests a crucial role for the JAK-STAT pathway in modulating serotonin receptor activities and influencing PLC signaling, providing insight into the neurobiological underpinnings of stress and mood disorders and potentially opening avenues for targeted therapeutic strategies.

The 5-HT2A receptor is widely distributed in peripheral tissues and is known to be coupled to Gαq, activating the signaling pathways of phospholipase C (PLC) and protein kinase C (PKC). Furthermore, the 5-HT2A receptor has been shown to activate the JAK2-STAT3 pathway along with the MEK-ERK1/2 pathway and the JAK2-STAT3 pathway, which are associated with survival, differentiation, migration, and invasion [166–168]. This indicates that the 5-HT2A receptor can activate multiple mitogenic pathways, including JAK2-STAT3 and PKC-Ras-Raf-1-MAPK, in various cell types [169]. Atypical antipsychotics, such as olanzapine, clozapine, and MDL100907, have been shown to induce desensitization of 5-HT2A receptor signaling and decrease phospholipase C (PLC) activity [162–165]. The mechanisms underlying this desensitization process are likely tissue-specific and involve multiple processes. In particular, activation of the JAK-STAT signaling pathway by atypical antipsychotics leads to increased expression of G protein signaling (RGS) protein regulators, particularly RGS7 [170]. This up-regulation of RGS7 expression is observed in both in vitro and in vivo models and contributes to desensitization of 5-HT2A receptor signaling by terminating activated Gαq/11 proteins more rapidly [164, 165]. Furthermore, drugs such as fluoxetine (an atypical antipsychotic) and paroxetine (a selective serotonin reuptake inhibitor, SSRI) have been shown to have anti-inflammatory effects by reducing serum concentrations of inflammatory cytokines IL-1β, IL-6, and TNF-α, and inhibiting the expression of the JAK-STAT3 and TLR4/JNK gene in macrophages [171, 172]. These effects of paroxetine are partially mediated by the 5-HT systems present in immune cells and the JAK2-STAT3 pathway, indicating a role for serotonin in immunosuppressive processes [171].

In summary, the interaction between serotonin and JAK, particularly JAK2, is involved in the desensitization of 5-HT2A receptor signaling by atypical antipsychotics. Activation of the JAK-STAT pathway is necessary for the complete desensitization response and up-regulation of RGS7 expression. Furthermore, modulation of JAK-STAT3 signaling by fluoxetine and paroxetine suggests their potential as anti-inflammatory agents [162–165, 171, 172]. These findings shed light on the complex interactions between serotonin receptors and the JAK-STAT pathway, providing information on the pathophysiology of psychiatric disorders and offering potential avenues for targeted therapeutic interventions.

Furthermore, IL-6 is considered a prominent example of a STAT3 activator that triggers the JAK-STAT pathway [173]. Although the membrane-bound IL-6 receptor (IL-6R) is expressed only in a few cell types, a soluble form of IL-6R (sIL-6R) can bind to extracellular IL-6, forming a complex with the ubiquitously expressed cell surface glycoprotein 130 (gp130) [174] (Fig. 1). This transsignaling mechanism allows IL-6 to target various cell types, including neurons and other types of CNS cells [174]. The consistently reported link between IL-6 activity and psychopathology is proposed to be primarily related to this type of signaling [175]. As one of the main downstream effectors of IL-6, the prominent role of STAT3 in psychopathology suggests a possible involvement of STAT3 signaling within the immune theory of psychiatric illness [176]. Evidence suggests that IL-6 can regulate serotonin transporter (SERT) expression and function, mediated by STAT3 activation, and systemic blockage of STAT3 leads to altered behavioral phenotypes relevant to mood disorders [177]. There are also data that demonstrate the regulation of STAT3 expression by SSRIs, which block SERT, suggesting a regulatory feedback loop between STAT3 and SERT expression [171]. These findings indicate that the effects of IL-6 on the serotonergic system and behaviors relevant to psychiatric disorders may be mediated by JAK-STAT3 [176].

In general, serotonin can regulate JAK-STAT3 activation in certain cell types, leading to downstream transcriptional effects dependent on serotonin and STAT3. Furthermore, there is evidence of molecular interactions between STAT3 and important elements of the serotonergic machinery. For example, lithium chloride, a mood stabilizer used in the treatment of bipolar disorder and treatment-resistant major depressive disorder, inhibits STAT3 activation in astrocytes and reduces JAK-STAT3 signaling activity [178, 179]. Knockdown of dorsal raphe (DR) STAT3 phenocopies behavioral alterations observed in STAT3 knockout mice, suggesting a role for STAT3 signaling in controlling behavioral reactivity relevant to psychopathology [180, 181]. Collectively, these results highlight the involvement of the JAK-STAT pathway in the interaction between serotonin receptors and psychiatric disorders, providing information on potential therapeutic targets and mechanisms underlying these conditions.

#### JAK-STAT and other neurotransmitter receptors

The JAK-STAT pathway has a multifaceted involvement in the regulation of neurotransmitter receptors, especially AMPA (α-amino-3-hydroxy-5-methyl-4-isoxazolepropionic acid receptors), NMDA (N-methyl-D-aspartate receptors), and muscarinic receptors, within the hippocampus, with implications for learning and memory [46]. For example, NMDA receptors, essential for excitatory transmission and synaptic plasticity in the CNS, interact with IL-6 in a JAK-STAT-dependent manner. Specifically, IL-6 elevates calcium influx through NMDA receptors, an action that can be counteracted by the NMDA antagonist MK-801; this process is mediated via the JAK-STAT pathway, as blocking JAK3 negates the IL-6-induced calcium influx [182].

In the realm of cholinergic neurotransmission, the JAK2/STAT3 signaling axis is essential. Inactivation of this pathway leads to a cascade of effects that include downregulation of choline acetyltransferase, an enzyme crucial for acetylcholine synthesis, and desensitization of M1-type muscarinic acetylcholine receptors. These effects combine to negatively influence spatial working memory [183, 184]. Further supporting the significance of the pathway, Colivelin, a humanin derivative, enhances ERK phosphorylation through a JAK2- and STAT3-dependent mechanism. Interference in this pathway, for instance through inhibition of JAK2, directly affects cholinergic function and spatial memory [181, 183, 184].

Collectively, these findings position the JAK-STAT pathway as a central player in modulating neurotransmitter receptors and associated cognitive functions, acting as an integral part of a complex regulatory network.

## JAK-STAT pathway under pathological conditions in the CNS

**Fig. 2:**
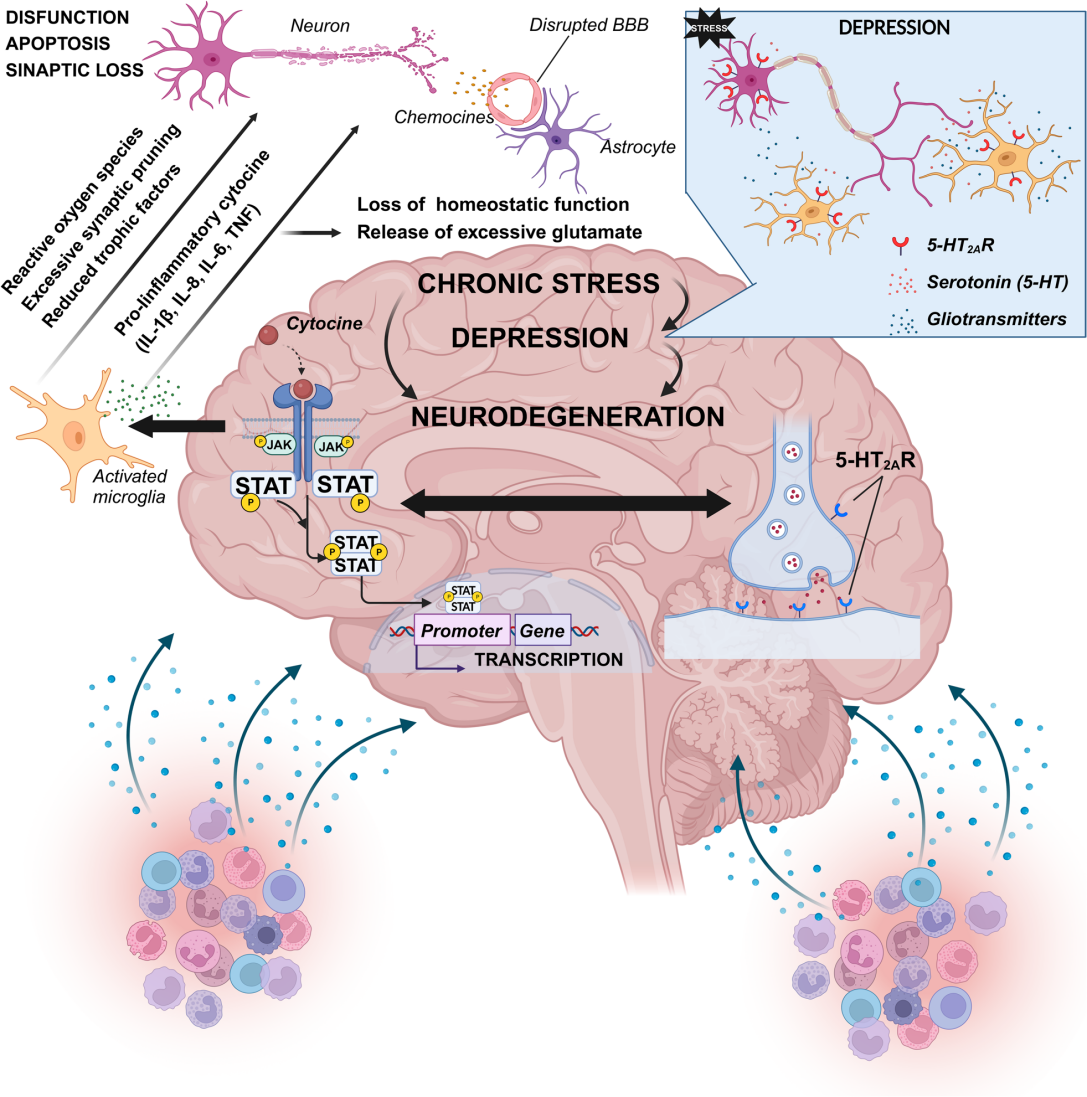
JAK-STAT pathway's role in pathological conditions. The figure offers a schematic representation of the JAK-STAT pathway's role in pathological conditions, prominently depicted in the left central portion. It underscores the pathway's association with chronic stress, depression, and neurodegenerative processes. Notably, the 5-HT2A receptor is known to activate several mitogenic pathways, including JAK2-STAT3 and PKC-Ras-Raf-1-MAPK, across various cellular configurations. Concurrently, data illustrate the regulation of STAT3 expression by selective serotonin reuptake inhibitors (SSRIs), which inhibit the presynaptic serotonin reverse transporter (SERT), suggesting a regulatory feedback nexus between STAT3 and SERT expression. Generally, serotonin is posited to modulate JAK-STAT3 activation in specific cell types, culminating in downstream transcriptional ramifications contingent on serotonin and STAT3. Further, the right central segment of the figure illuminates molecular interactions between STAT3 and pivotal components of the serotonergic machinery. These insights accentuate the JAK-STAT pathway's role in the interface between serotonin receptors and psychiatric afflictions, offering valuable information about prospective therapeutic targets and the underlying mechanisms of these disorders. It is pivotal to highlight that such interactions against a backdrop of stress are accompanied by an elevated concentration of pro-inflammatory cytokines in the peripheral blood, attributed to the activation of immune cells, indicating chronic low-grade systemic inflammation. This interaction is depicted in the bottom left and right sections of the figure. A consequential impairment in the Blood–Brain Barrier (BBB) function leads to microglial activation and the subsequent release of pro-inflammatory cytokines, as presented in the left central segment. This cascade culminates in compromised neuronal functionality

CNS inflammation refers to the activation of immune responses within the CNS, characterized by the recruitment and activation of immune cells, the release of pro-inflammatory mediators, and the disruption of normal cellular functions (Fig. 2). It can occur from various causes, including infection, autoimmune disorders, neurodegenerative diseases, and psychological stressors such as depression. The key players in CNS inflammation are microglia and astrocytes, which get activated and release factors such as cytokines, chemokines, and growth factors. This leads to complex cross-talk between different types of brain cells, including microglia, astrocytes, neurons, and endothelial cells. Increased expression of inflammation-associated genes characterizes the inflammatory locus.

### Role of STAT isoforms in the development of neuroinflammation

The JAK-STAT signaling pathway is involved in the regulation of inflammation in the CNS. Dysregulation of this pathway has pathological implications for neuroinflammatory diseases, particularly in triggering and polarizing myeloid cells and T cells toward pathogenic phenotypes [185]. The activation of the JAK-STAT pathway is influenced by different cytokines. For example, anti-inflammatory cytokines such as IL-10 activate STAT3, while pro-inflammatory cytokines like IFN-γ induce STAT1 phosphorylation through JAK2 [186]. The JAK-STAT transduction pathway is a highly conserved signaling cascade that intricately regulates cellular processes such as proliferation, differentiation, survival, and apoptosis. Perturbations within this pathway are closely related to the emergence of metabolic and neurological disorders. Recently, the exploration of neuroinflammatory agents has generated significant research interest [187–207]. Within the STAT family of transcription factors, comprising six main isoforms (STAT1, STAT2, STAT3, STAT4, STAT5, STAT6) [154, 187], emerging scientific insights affirm the distinct roles of individual STAT members in the pathogenesis of neuroinflammation (Table 4).

**Table 4:** Role of STAT isoforms in the development of neuroinflammation

STAT isoform	Role in neuroinflammation
STAT1	• Activation of STAT1 triggers microglial polarization toward M1, as evidenced by Butturini et al. in 2019 [189] • Neuroinflammatory diseases activate the STAT1 / ASPP2 pathway in astrocytes, as demonstrated by Turnquist et al. in 2014 [190] • The miRNA-338-3p / STAT1 pathway suppresses pro-inflammatory cytokines in astrocytes and microglia, as elucidated by Liu et al. in 2023 [191] • The JAK2 / STAT1 pathway participates in the pro-inflammatory microglial phenotype, indicated by Zang et al. in 2022 [192] • Abrocitinib inhibits JAK1/STAT1/NF-κB pathway, dampening microglia-mediated neuroinflammation, by Li et al. in 2022 [193] • Curcumin downregulates the JAK1 / STAT1 pathway, improving brain damage and M1 microglia polarization in stroke-induced neuroinflammation, Wang et al. in 2022 [194]
STAT2	• STAT2 in CNS astrocytes influences the immune-glial interaction after CNS injury, by Khorooshi et al. in 2008 [195] • STAT2 extends to nonviral infection responses, forming a STAT1 / STAT2 heterodimer, as stated by Rauch et al. in 2013 [196]
STAT3	• STAT3 depletion shifts microglial polarization from M1 to M2, after subarachnoid hemorrhage, Zheng et al. in 2022 [197] • Stattic inhibits STAT3, reducing 193-induced microglial activation in the hippocampus of mice, Millot et al. in 2020 [198] • In 2021, Hu et al. find an increase in STAT3 expression in neuroinflammation related to experimental periodontitis in rats [199]
STAT4	• The potential role of STAT4 in neuroinflammation involves the participation of T cells [200]
STAT5	• The JAK2/STAT5 pathway plays a pivotal role in IL-3-induced microglial activation [201] • The anti-inflammatory impact of dauricin on microglia is largely mediated by the STAT5/NF-κB pathway, possibly acting as a STAT5 inhibitor [202] • In an experimental model of autoimmune encephalitis, STAT5 tetramers are instrumental in driving Th17 chemotaxis into meninges through the GM-CSF-STAT5-CCL17 mechanism. These tetramers present promising targets for targeted therapy [203] • The presence of STAT5 is critical in autoimmune neuroinflammation, being necessary for the pathogenicity of T-helper cells, particularly linked to hyperproduction of GM-CSF [204] • The role of STAT5 in the formation of autoreactive CD4 + T cells in the context of neuroinflammation is confirmed not only by the research of Lawson BR (2015), but also by other authors [205]
STAT6	• IL-4 induces the shift of microglial polarization from the M1 to M2 state by activating the JAK1/STAT6 pathway, which ultimately contributes to alleviation of neurological damage [206] • Electroacupuncture improves the polarization of M2 microglia and the anti-inflammatory response of the hippocampal glia in Alzheimer's disease. This effect is partially mediated by STAT6 activation [207] • Astragalin promotes the shift of microglial polarization towards the M2 phenotype, leading to alleviation of depressive symptoms. This process involves the prevention of STAT6 degradation [208] • Inhibition of the IL-4Rα/JAK1 and JAK3/STAT6 pathways results in polarization of microglia toward the M1 phenotype during the development of neuroinflammation [209]

In this context, STAT1 plays a pivotal role in steering the polarization of microglial stromal macrophages towards the pro-inflammatory M1 pole (Table 4). It's noteworthy that STAT1 also takes center stage in orchestrating the type 1 immune response (i1), characterized by heightened macrophage and effector cell activity [90]. Meanwhile, STAT2 predominantly mediates astrocyte activation, particularly in viral neuroinfections. Directly involved in the pro-inflammatory activation of microglia and astrocytes in various neuroinflammatory scenarios, STAT3 assumes a critical role, while STAT4 indirectly activates these cells by enlisting T cells in the inflammatory fold (Table 2). The JAK2/STAT5/NF-κB pathway intricately engages microglial activation and Th17 helper cell participation in neuroinflammation, particularly in immune reactions to autoantigens. On the contrary, STAT6 promotes microglial differentiation towards the functional M2 pole, indicating the resolution of neuroinflammation (Table 4). Evidently, the potential modulation of specific STAT isoforms holds promise for fine-tuning the trajectory of neuroinflammation development. It is an intriguing avenue for targeted therapies that could address the intricate interplay between distinct STAT proteins and their implications in neuroinflammatory cascades.

### Role of JAK-STAT in demyelinating and neurodegenerative diseases (multiple sclerosis and Parkinson's disease)

Chronic inflammation in the CNS has long been observed in Parkinson's disease (PD) and has been proposed to play an important role in the pathogenesis of the disease [95]. Suppression or modulation of the JAK-STAT pathway has shown promise in preclinical studies as a potential therapeutic strategy for PD and other neuroinflammatory diseases [95]. Inhibition of the JAK-STAT pathway influences both innate and adaptive immune responses by suppressing α-SYN-induced microglia activation and CD4 + T cell recruitment to the CNS, ultimately suppressing neurodegeneration (Fig. 2). The findings of Qin H et al. (2016) were the first documentation that suppression of the JAK-STAT pathway disrupts the circuitry of neuroinflammation and neurodegeneration, thus attenuating the pathogenesis of PD [187]. Until now, a variety of synthetic or natural small molecule Jakinibs have shown promise in the treatment of a variety of diseases, many of which are in preclinical research or clinical trials [210, 211].

Although the involvement of JAK-STAT signaling in the pathogenesis of multiple sclerosis (MS) and its animal model, experimental autoimmune encephalomyelitis (EAE), is known, the underlying mechanisms are complex due to the involvement of multiple cellular components. In EAE, autoreactive Th1 cells produce IFN-γ via STAT4, promoting the activation of pro-inflammatory macrophages through STAT1. Similarly, Th17 cells produce GM-CSF in the CNS, favoring pro-inflammatory polarization of macrophages through JAK2/STAT5 [212]. Pro-inflammatory microglia/macrophages facilitate Th1 polarization through JAK2/TYK2 and STAT4 by producing IL-12, while they facilitate Th17 polarization through JAK1/2 and STAT3 by producing IL-23 and IL-6. Infiltrating neutrophils contribute to brain inflammation in EAE and are critical for the occurrence of the atypical phenotype of EAE. G-CSF, produced during EAE, promotes neutrophil differentiation and activation through JAK1/2 and STAT3 [213]. Astrocyte activation in response to ER stress occurs through JAK1/STAT3 signaling, and STAT3 activation depends on PERK, a central sensor of ER stress. ER stress-activated astrocytes secrete pro-inflammatory cytokines such as IL-6 and oncostatin M (OSM) [213]. IL-6 produced by astrocytes has been shown to be critical for EAE induction, and IFN-γ signaling in astrocytes is involved in EAE [92]. Astrocytes also produce GM-CSF during the process of EAE disease, polarizing microglia and infiltrating monocytes into the pro-inflammatory phenotype via JAK2/STAT5 [96]. Modulation of JAK-STAT signaling appears to play a role in chemical-induced demyelination, suggesting its involvement in the promotion of oligodendrocyte apoptosis and demyelination [214]. In Alzheimer's disease (AD), neuroinflammation, along with the presence of Aβ plaques and neurofibrillary tangles, is a fundamental neuropathological characteristic. The JAK-STAT signaling pathway associated with neuroinflammation has been evaluated as a target for drug therapy in AD. However, there are limited data available on the modulation of the JAK-STAT signaling pathway in the development of AD. Research is currently focused on understanding the complex pathophysiology of AD, including aberrant protein metabolism, inflammatory responses, and other processes, to identify potential therapeutic strategies [214].

### Role of JAK-STAT during oxidative stress in the CNS

Another potential mechanism that involves JAKs is oxidative stress in the CNS. Oxidative stress occurs when the production of free radicals exceeds the antioxidant capacity of the CNS. Modern molecular pathophysiology studies have confirmed the significant role of oxidative stress in various pathological changes in the CNS, including hypoxic/toxic injury, metabolic disturbance, inflammation, and oncogenesis (Fig. 2) [215–219]. Although ROS were previously considered harmful substances that could cause cell damage and lead to various pathological processes in the CNS, they are now recognized as important signal molecules that regulate various activities of the CNS [215]. Therefore, on the one hand, disruption of neurotrophic factor signaling by oxidative stress mediators can contribute to neuronal damage observed in neurodegenerative diseases and significantly affect the therapeutic efficacy of ciliary neurotrophic factors (CNTFs) in preventing nerve cell death [216]. The action of CNTF signaling is predominantly mediated by the activation of Jak1/Jak2 [217]. Although the levels of CNTF-like factors and CNTF receptor subunits appear unchanged in neuroinflammatory diseases, their activities can be altered, contributing to loss of neuronal function. In nerve cells, exposure to agents that increase oxidative stress results in blockade of the JAK-STAT pathway and disruption of growth factor and cytokine signaling, which could contribute to neurodegenerative damage [216]. Furthermore, oxidative stress-induced disruption of STAT survival signaling in neurons, combined with enhanced STAT inflammatory signaling in non-neuronal cells, can further amplify neural injury. Furthermore, microglia activation triggers the activation of downstream kinases and transcription factors (JNK, p38 MAPK, JAK-STAT and NF-κB) [218]. Subsequently, these factors can upregulate immune molecules such as iNOS and NADPH oxidase (NOX), leading to the generation of NO and superoxide, respectively. Thus, specifically targeting the JAK-STAT and NF-κB signaling pathways may have potential as anti-inflammatory strategies to confer cerebrovascular protection [208].

Increasing evidence has revealed the crucial role of astrocytes in the regulation of oxidative stress in the CNS [219]. The JAK-STAT signaling pathway emerges as a key mediator of astrogliosis [220]. Activated astrocytes exhibit dual properties known as A1 and A2 astrocytes. A1 astrocytes promote neuronal loss by inducing inflammation through the NF-kB pathway, which affects neuronal protection and synaptogenesis control [221]. In contrast, A2 astrocytes promote neuronal survival through the Janus kinase/signal transducer and activator of the JAK-STAT3 signaling pathway by upregulating neurotrophic factors [222].

In summary, the JAK-STAT pathway plays an important role during oxidative stress in the CNS, influencing neurotrophic factor signaling, neuronal damage, and inflammatory responses. Furthermore, activated astrocytes exhibit ambidextrous properties that can contribute to neuronal loss or promote neuronal survival, depending on the specific astrocyte phenotype.

### BDNF dysregulation

Brain-derived neurotrophic factor (BDNF) is involved in various crucial processes in the brain, including plasticity, neuronal survival, synapse formation, dendritic branching, and modulation of neurotransmitter profiles [223]. Dysregulation of BDNF release is commonly observed in brain pathologies, leading to reduced levels of BDNF in the brain and blood [224]. Studies have revealed associations between low levels of BDNF or high inflammatory markers and the development of depressive symptoms, highlighting the bidirectional regulation between these factors and their implications for depression and the antidepressant response [224, 225].

BDNF activation of its receptors modulates multiple signaling pathways, such as MAPK/ERK, PLCγ, PI3K, JNK, and NFkB [226]. In particular, increased levels of BDNF have been shown to activate JAK-STAT in both in vivo and in vitro models, including the rat model of epilepsy and primary cultured neurons [227]. Deep RNA sequencing studies have also demonstrated that BDNF-induced JAK-STAT signaling is enriched with genes involved in synaptic neurotransmission, including ion channels, neurotransmitter receptors, and regulators of synaptic plasticity, neurogenesis, transcriptional regulation, neuroinflammation, and proliferation [228]. Interestingly, the mechanism of BDNF-induced regulation of JAK-STAT appears to be noncanonical, as STAT3 phosphorylation in Tyr705 is unlikely to control genome expression in neurons [228].

In summary, dysregulation of BDNF is associated with various brain pathologies and the development of depressive symptoms. BDNF activation of its receptors influences multiple signaling pathways, including the JAK-STAT pathway, which plays a role in synaptic neurotransmission and various cellular processes involved in brain function and pathology.

## Role of JAK-STAT in stress response and stress-associated diseases

The stress response and stress-associated diseases can be classified under both physiological and pathological conditions, depending on factors such as duration, intensity, and impact. In physiological settings, the acute stress response is often adaptive, activating the "fight-or-flight" mechanism to cope with immediate challenges and usually resolving upon the removal of the stressor. This serves the broader purpose of maintaining homeostasis. Conversely, under pathological conditions, prolonged exposure to stress leads to chronic activation of these physiological responses, resulting in deleterious effects such as chronic inflammation, compromised immune function, and increased susceptibility to diseases such as cardiovascular disorders and autoimmune diseases. Furthermore, maladaptive stress responses, exemplified by conditions such as post-traumatic stress disorder (PTSD), indicate instances where the stress mechanism itself becomes pathological. Therefore, the stress response and its associated diseases can manifest across a spectrum of physiological to pathological conditions, depending on the complexities of the stressors involved. Thus, the intricacy of stress phenomena justifies their segregation into a dedicated chapter.

The relationship between inflammation and depression/stress is bidirectional and complex [5–7]. Inflammation can contribute to the development of depressive symptoms, while depression and stress can lead to increased inflammation [229–235]. The mechanisms underlying this relationship are still being investigated, providing an opportunity to explore the non-classical actions of JAK kinases and their interactions with receptors, including serotonergic receptors [229–260]. The significant involvement of the JAK-STAT pathway in the stress response is underscored by research demonstrating up-regulation of genes governed by inflammatory transcription pathways, such as NF-κB and JAK-STAT, after exposure to acute stress [235]. A substantial proportion of genes within the JAK/STAT signaling pathways exhibited a pattern of up-regulated gene expression from baseline to stress, followed by down-regulation during the recovery phase [235].

### JAKs in stress response

Stress exposure triggers a cascade of hormonal, neurotransmitter, and immune responses that aim to maintain homeostasis. The activation of the sympathetic nervous system, which releases catecholamines such as adrenaline and noradrenaline, is a crucial component of the stress response. Recent studies suggest that these hormones can also indirectly influence the JAK-STAT pathway [230, 231]. Despite recent advances, a comprehensive understanding of the intricate interactions between catecholamines, JAK-STAT, and their roles in the stress response and associated pathologies remains elusive. The ongoing research aims to unravel these complex mechanisms and shed light on the interaction between catecholamines and JAK-STAT in the stress response. Adrenaline and noradrenaline indirectly impact the JAK-STAT pathway, primarily through the release of cytokines, particularly IL-6. Cytokines significantly affect the adrenal medulla, leading to changes in secretion, intracellular signaling, gene transcription, and translation [61, 231–236]. IL-6 activation has been reported to involve the JAK-STAT3 and MAPK/ERK signaling pathways, further highlighting the interconnectedness of these signaling mechanisms [233]. In turn, adrenergic receptors, when activated, can stimulate various signaling cascades, including the PI3K/Akt pathway, which regulates cell survival and function. The PI3K/Akt pathway interacts with JAK-STAT and can modulate its activity [232]. Thus, mental disorders can arise from inhibited PI3K/Akt signaling, affecting the function of neuronal and hippocampal stem cells. However, chronic stress can activate PI3K/Akt signaling through glucocorticoids and binding of noradrenaline to β-adrenergic receptors [231].

#### JAKs and the HPA axis activation

Combined with the sympathetic response, activation of the hypothalamic–pituitary–adrenal (HPA) axis leads to the release of cortisol, the primary stress hormone. Cortisol plays a crucial role in maintaining homeostasis and also acts as an immune suppressor to prevent excessive immune system activation during stress [234–239].

The activation of the cortisol-mediated glucocorticoid receptor (GR) exerts broad anti-inflammatory effects by inhibiting NF-κB/Rel transcription factors and other pro-inflammatory signaling pathways, including the JAK-STAT pathway [234]. Cortisol has also been shown to suppress cytokine expression, such as IL-1β, IL-8, IL-6, and TNFα2, in various cell types in response to immunostimulants and lipopolysaccharide (LPS) [236–240]. LPS, recognized by TLR4, activates downstream signaling through the NFκB and JAK-STAT pathways [236, 238, 241]. Negative regulation of cortisol and growth hormone (GH) signaling in mammals is mediated by SOCS genes, which target the JAK-STAT pathway [242–244]. This regulation by SOCS may integrate various physiological functions related to the redistribution of energy substrates [242]. In particular, a study of trout by Philip AM and Vijayan MM (2015) suggests that cortisol-induced upregulation of SOCS-1 and SOCS-2 transcript levels, leading to a reduction in the JAK-STAT signaling pathway, may represent a novel mechanism that contributes to growth reduction and immune suppression during stress [243].

Under normal conditions, leptin signals satiety and regulates energy balance, while stress leads to increased cortisol and decreased leptin levels [238, 239]. Although it is primarily associated with satiety, leptin plays multiple roles in energy homeostasis, metabolism, exercise, and neuroendocrine function [224, 225]. Reduced leptin levels are associated with hyperphagia, hypogonadotropic hypogonadism, suppressed levels of thyroid and growth hormone (GH), and affect glucose homeostasis independently of food intake [244–247]. Leptin displays pulsatile and diurnal patterns, peaking around midnight and inversely correlating with pituitary-adrenal function. Glucocorticoids raise leptin levels, partially independent of changes in fat mass, while activation of the ACTH or HPA axis does not appear to acutely affect plasma leptin [248]. Acute stress rapidly decreases leptin levels along with increased cortisol, suggesting leptin as a potential stress biomarker [244, 245, 249]. Chronic stress reduces leptin levels and offers hypotheses: decreased leptin production may contribute to depression-like manifestations, the hippocampus may mediate antidepressant-like effects of leptin, and enhanced brain leptin signaling might aid in the treatment of depressive disorders [250]. Leptin administration, systemically or directly to the brain, counteracts depression-related deficits related to chronic stress [250, 251]. Leptin also affects immunity, increasing the activation, chemotaxis, and survival of innate and adaptive immune cells [252]. By engaging the JAK-STAT pathway through its receptor, the various actions of leptin underscore its crucial role in metabolic, stress, and immune responses [253].

In summary, cortisol, the primary stress hormone, plays a pivotal role in the regulation of physiological processes and immune responses during stress. It exerts anti-inflammatory effects by inhibiting pro-inflammatory signaling pathways, including JAK-STAT. Leptin, in conjunction with cortisol, participates in metabolic and stress responses, while also affecting immune function through JAK-STAT signaling. These interactions highlight the complex interaction between hormones, neurotransmitters, and the JAK-STAT pathway in the stress response and its implications for various physiological and pathological conditions.

#### Serotonin, growth hormone and JAKs

Growth hormone (GH) plays a pivotal role in stress responses, delicately balancing with cortisol. Stress triggers increased GH secretion, which acts as a cortisol counterregulatory hormone, conserving muscle mass and maintaining energy by promoting protein synthesis and lipolysis. GH also engages the immune system, improving pro-inflammatory cytokine production, mobilizing immune cells, and increasing the activity of natural killer cells. These actions underscore the role of GH in orchestrating an efficient immune response during stress. Furthermore, GH contributes to energy balance by reducing insulin sensitivity and promoting gluconeogenesis, ensuring steady glucose supply to vital organs, including the brain [254]. GH signals primarily through the JAK-STAT pathway. GH binding to its receptor (GHR) initiates the JAK-STAT cascade, culminating in the transcription of the target genes, including insulin-like growth factor 1 (IGF-1) [243]. However, immunostimulants such as lipopolysaccharide (LPS) negatively regulate GH signaling in mammals by altering STAT5 activation and JAK-STAT signal transduction [243, 255]. This intricate GH-mediated network ensures a robust stress response, highlighting its vital role in protecting health during challenging situations.

Serotonin activity can increase or decrease depending on the nature, intensity, and duration of the stressor. Acute stress often leads to an immediate increase in serotonin release in various regions of the brain, influencing mood and anxiety. On the contrary, chronic stress is commonly associated with a decrease in serotonin levels, which contributes to the development of depression and anxiety disorders [256–260]. The interaction between serotonin and cortisol adds another layer of complexity to the stress response, particularly in relation to the immune system. Therefore, serotonin not only acts as a mood regulator, but also functions as a growth factor for specific immune cells and modulates the release of cytokines, influencing the intensity and trajectory of the immune response. The interaction between the JAK-STAT pathway and the brain neurotransmitter system likely occurs through cytokines. In particular, IL-6 has been found to directly regulate serotonin transporter (SERT) levels, which impact serotonin reuptake. This regulation, dependent on STAT3, provides a potential neurobiological basis for the involvement of IL-6 in depression [258]. Therefore, studying the interconnected pathways of serotonin, cortisol, and JAK-STAT may provide valuable information on the biological mechanisms underlying stress responses and mood disorders.

In conclusion, the JAK-STAT pathway plays a crucial role in the stress response and stress-related diseases. It is involved in the bidirectional relationship between inflammation and depression/stress and interacts with neurotransmitter systems such as serotonin. The activation of JAK-STAT signaling is observed in response to stressors, and its dysregulation is implicated in neuropsychiatric disorders. Hormones such as cortisol and growth hormone, which are released during stress, modulate the JAK-STAT pathway to maintain homeostasis and coordinate immune responses. The recovery phase after stress involves the recalibration of the JAK-STAT pathway along with other physiological processes. More research is needed to fully understand the intricate interactions between the JAK-STAT pathway, stress neurotransmitters, and their implications for health and disease.

### JAKs in stress-associated diseases

The JAK-STAT signaling pathway is integral to a myriad of cellular processes including inflammation, neurogenesis, and synaptic plasticity. Recent research has illuminated its critical involvement in a variety of neuropsychiatric conditions [259–266]. For example, studies have observed a pronounced increase in JAK3 expression coupled with a decrease in STAT1 expression in patients with depressive disorders compared to healthy controls [259, 260]. These observations lend credence to the hypothesis that a dysregulated JAK-STAT signaling pathway may be involved in the pathogenesis of depressive conditions. Gulbins et al. (2016) conducted a seminal study illustrating the impactful role of JAK3 in stress-induced behavior and hippocampal neurogenesis [259]. They found that stress leads to the activation of JAK3, which in turn inhibits neurogenesis and induces depressive-like behavior. Direct inhibition of JAK3 significantly mitigated these adverse effects. Furthermore, it was shown that the mechanism through which JAK3 becomes activated in response to stress involves acid sphingomyelinase. Administration of amitriptyline, an antidepressant drug, resulted in a reduction in JAK3 phosphorylation and an improvement in both behavior and hippocampal neurogenesis.

The mature nervous system employs self-protective mechanisms in response to sublethal stressors, including the release of growth factors and cytokines, as well as the activation of intracellular signaling pathways. Here, ciliary neurotrophic factor (CNTF) emerges as a key player in neuroprotection, activating both the JAK-STAT and ras-MAPK cascades [232]. A case–control study by Elouaer et al. (2023) added another dimension to understanding the role of JAK-STAT by exploring its association with bipolar disorder [260]. Although no conclusive links were established between specific JAK1 gene polymorphisms and the susceptibility to bipolar disorder, a significant correlation was found with elevated psychiatric rating scores among patients with a certain genotype. Further complicating this landscape is the research by Long et al. (2023), which demonstrated that minocycline administration in combination with antipsychotics effectively inhibited microglial activation, implicating MAPK and JAK-STAT pathways as mediators of anti-inflammatory and neuroprotective effects [261]. These findings are particularly noteworthy for their implications in improving the negative symptoms associated with schizophrenia. Another study by Almutabagani et al. (2023) revealed the dysregulation of the JAK2/STAT3 signaling pathway as a significant contributor to treatment-resistant depression, suggesting that targeting this inflammatory component could increase the therapeutic efficacy of existing antidepressant regimens [262]. On a different note, Lee et al. (2016) postulated that JAK-STAT signaling could be involved in the neurogenic-to-gliogenic shift observed in Down Syndrome [263]. Overexpression of interferon receptors in HSA21 was suggested to modulate JAK-STAT signaling, thus affecting brain development in affected individuals. Adding to the therapeutic potential of manipulating JAK-STAT signaling, a study by Borbély et al. (2022) found that inhibitors targeting this pathway ameliorated stress-induced reductions in adult neurogenesis and alleviated associated anxious-depressive behavior in murine models, leading to the initiation of a Phase I/II clinical trial aimed at assessing these inhibitors in human patients diagnosed with treatment-resistant depression [264].

In sum, the body of evidence accumulated thus far underscores the critical implications of the JAK-STAT signaling pathway in the etiology and treatment of neuropsychiatric disorders. These insights not only deepen our understanding of the mechanistic underpinnings but also open new avenues for innovative therapeutic strategies.

## Therapeutic developments and opportunities: JAK-STAT inhibition

### Clinically applied Jakinibs

Currently, a variety of inhibitors targeting the JAK-STAT pathway are being applied clinically, primarily for a specific set of diseases, including rheumatoid arthritis, canine dermatitis, psoriasis, ulcerative colitis, myelofibrosis, polycythemia vera, and primary thrombocytosis (as reviewed in [267–271]). Importantly, it should be emphasized that the scope of the diseases investigated is relatively narrow. These conditions share predominantly commonalities in their pathogenesis, characterized in part by dysregulated immune responses or inflammatory processes. Therefore, clinical trials are largely focused on these disorders with similar pathological key points.

According to the Clinical Trials Registry (https://clinicaltrials.gov/), at June 23, there are 143 listed studies, of which 61 have been completed and 15 have been terminated (the entire list of studies can be found as Supplementary Table S1 online). There are 12 active clinical trials in Phase 2 and 3. Currently, drugs under investigation include baricitinib for patients with Aicardi Goutières syndrome (AGS) and adult idiopathic inflammatory myositis (IIM) [ClinicalTrials.gov ID NCT04208464], Ruxolitinib for patients with vitiligo (ClinicalTrials.gov ID NCT04896385), atopic dermatitis (ClinicalTrials.gov ID NCT05456529), B-cell acute lymphoblastic leukemia B cells in conjunction with chemotherapy (ClinicalTrials.gov ID NCT02723994), chronic chronic cutaneous graft-versus-host disease (ClinicalTrials.gov ID NCT03954236), and to test the efficacy of a JAK inhibitor prior to a donor stem cell transplant in treating patients with myelofibrosis (ClinicalTrials.gov ID NCT02251821). Itacitinib is being studied in combination with corticosteroids as a first-line treatment for moderate or severe chronic graft versus host disease (cGVHD) (ClinicalTrials.gov ID NCT03584516), while PF-06651600 is under investigation for alopecia (ClinicalTrials.gov ID NCT04006457), and Momelotinib for patients with symptomatic and anemic myelofibrosis (ClinicalTrials.gov ID NCT04173494). These data indicate a focused exploration of the JAK-STAT pathway within a constrained set of conditions, underscoring the need for expanded research into the potential applicability of these inhibitors in a broader spectrum of diseases.

### Novel applications for Jakinibs

Emerging research on Jakinibs has illuminated their versatile therapeutic potential beyond their established applications in immunological and inflammatory maladies. Seminal studies by Gulbins et al. (2016) have delineated the role of JAK3 activation in stress-induced neurogenesis impairment and anxious-depressive behavior in animal models, thus establishing the foundation for treating major depressive disorder [259]. Simultaneously, a 2021 meta-analysis showcased the improvement of mental health outcomes in rheumatoid arthritis patients when Jakinibs were used either singularly or in conjunction with methotrexate [272]. These findings accentuate the multifaceted role of the JAK-STAT pathway in human health and require further nuanced assessment methodologies for mental health outcomes.

Despite these promising developments, obstacles remain to achieving optimal specificity and targeted drug delivery (Table 5). Noteworthy advances include the development of isoform-specific inhibitors such as filgotinib and upadacitinib aimed at mitigating off-target effects [273–275]. Incorporation of monoclonal antibodies such as adalimumab in combination treatments offers another layer of specificity [276].

**Table 5:** Strategic Directions for Advancing JAK Inhibitor Therapies in Inflammatory and Stress-Related Disorders: A Focus on Combination Approaches

Category	Findings/Current Applications	Future Research Directions and Considerations	References
Specificity	Isoform-specific inhibitors such as filgotinib and upadacitinib	Development of targeted cotherapies that leverage the specificity of Jakinibs alongside other agents	[274, 277, 278]
	Liposomal formulations for targeted drug delivery	Use of targeted release systems that deliver both Jakinibs and complementary agents to specific cells or tissues	[279, 280]
Therapeutic Efficacy	Efficacy in treating inflammatory conditions such as rheumatoid arthritis	Further research into the synergistic effects of Jakinibs with SSRI or benzodiazepines in stress disorders	[272, 273]
	Combination with methotrexate or monoclonal antibodies	Assessment of Jakinibs in combination with anti-inflammatory agents like corticosteroids or with neurotrophic factors like BDNF in stress disorders	[276]
	Biomarker-guided therapies in cancer	Development of precision medicine approaches that tailor combination therapies based on individual patient profiles	[281, 282]
Safety and Side Effects	Immunological concerns such as immunosuppression	Detailed pharmacokinetic studies to determine optimal dosage regimes that minimize adverse effects when using combination therapies	[283]
Regulatory Aspects	FDA-approved for specific conditions	Multiphase clinical trials to assess the safety and efficacy of combinatorial therapies with the aim of regulatory approval	

Innovative delivery mechanisms are also being implemented to address these challenges. Among them, nanoparticle-based systems such as liposomal formulations are used to enhance drug targeting [279]. Specialized administration routes, such as intranasal and intrathecal applications, are especially promising for conditions that require localized intervention. Intranasal ketamine, for example, quickly alleviates symptoms of treatment-resistant depression [284–288], while intrathecal injections have been proven to be effective in managing spasticity in multiple sclerosis, with minimal systemic exposure [288, 289]. Biomarker-guided strategies, such as those used in trastuzumab cancer therapies, add another layer of precision by utilizing HER2 status for drug delivery [277].

In the context of the COVID-19 pandemic, Jakinibs have garnered attention for their potential utility in treating SARS-CoV-2-induced conditions, such as hyperinflammation and fibrosis, as well as severe symptoms such as pneumonia and acute respiratory distress syndrome [90, 290–292]. Baricitinib has emerged as a strong candidate; however, the optimal time for its initiation remains to be determined. Selective JAK2 inhibitors, such as pacritinib and lestaurtinib, offer the advantage of not interfering with type I interferon signaling, essential for antiviral immunity [90, 293].

However, the deployment of Jakinibs is not without risks, including immunosuppression and potential hematopoietic deficiencies [272, 294–297]. As a result, their clinical utility requires a balanced strategy that rigorously evaluates safety, mandates vigilant monitoring, and comprehensively understands their biological mechanisms. In summary, to unlock the full therapeutic range of Jakinibs for various medical conditions, including neuropsychiatric and aging-related disorders, focused efforts are needed to overcome the challenges related to specificity and targeted drug delivery.

### Expanding the spectrum of JAK kinase inhibitors: a pathophysiological rationale

The intricate interplay between neurotransduction mechanisms and pro-inflammatory signaling pathways, exemplified by the JAK-STAT pathway at the cellular level, extends to the CNS and to the entire organism, playing a critical role in the relationship between neuroinflammation and stress-associated neuropsychic pathologies. To fully grasp the importance of these relationships, it is essential to define the concept of inflammation in this context. Recent advances in molecular biology and pharmacology have expanded our understanding of cellular stress and inflammation as a general pathological process [13, 14]. Traditional inflammation, characterized by a local tissue reaction to damage, is typically considered a response involving an exudative vascular reaction and leukocyte migration. However, systemic inflammation and hyperinflammation can develop as a result of classical inflammation or as a direct complication of certain diseases, such as COVID-19 [90, 187, 292].

Evolutionary canonical inflammation, associated with the progressive system of blood microcirculation and the emergence of the classical variant of the adaptive immune system in vertebrates, is related to the JAK-STAT signaling pathway. This pathway arose earlier, even before the separation of protostomes and deuterostomes in invertebrates, and is associated not only with canonical inflammation but also with more archaic types of inflammation and immunity [30, 298]. In vertebrates, the components of the JAK-STAT pathway diversified concurrently with the expansion of cytokines and cytokine receptors [272]. Low-grade chronic inflammation, predominantly associated with metabolic damage factors (meta-inflammation) and tissue aging (inflamm-aging), represents another variant of inflammation [298–300]. This form of inflammation involves various cells, including resident cells, stromal macrophages, connective tissue cells, and even brain cells, such as neurons, contributing to the formation of a cytokine network during neurogenic stress, pain, and mental illnesses [27, 301–303]. Pro-inflammatory cellular and tissue stress can play an adaptive role during the development of extreme physiological processes. However, low-grade chronic inflammation lacks the typical barrier function seen in traditional inflammation, making it easily delocalized. This type of inflammation is commonly associated with pathologies such as morbid obesity, metabolic syndrome, and type 2 diabetes mellitus [304–307].

It should be noted that the highly conserved JAK-STAT signaling pathway, necessary for normal homeostasis, can contribute to the development of low-grade chronic inflammation-associated tissue atrophy, such as skeletal muscle atrophy (sarcopenia) [38]. In turn, this contributes to further disruption of metabolic cycles involving muscles, liver, and adipose tissue [308, 309]. The reciprocal relationship between depression and systemic manifestations of low-grade chronic inflammation can form a vicious pathogenetic circle, strengthening each other [39, 40, 310–312]. Furthermore, local low-grade chronic inflammation can transform into classical inflammation, as observed in diabetic kidney disease [17]. Atherosclerosis demonstrates the characteristics of classical and low-grade chronic inflammation, indicating the possibility of escalation in the pro-inflammatory process and the emergence of various forms of neuroinflammation [17]. However, the transition from pro-inflammatory stress to neuroinflammation should be confirmed by the presence of neurodegeneration or other morphological signs of inflammation.

Neurodegenerative diseases, including Alzheimer's disease and Parkinson's disease, often exhibit low-grade chronic neuroinflammation manifestations [313, 314]. Peripheral inflammation, such as cytokinemia and increased intestinal permeability to neurotoxins, can also influence the development of depression and other stress-associated pathologies [315, 316]. Chronic stress contributes to oxidative damage in the colon and triggers immune responses involving mast cells, neutrophils, and monocytes, further highlighting the reciprocal relationship between inflammation and neuropsychiatric pathologies [317–319].

It is important to note that pro-inflammatory mechanisms are involved not only in traditionally considered inflammatory diseases such as cancer but also in various physiological processes. These processes include normal immunogenesis, maintenance of integumentary tissue barrier functions, and skeletal muscle function [320]. Even under physiological conditions, these tissues experience damaging factors and potentially dangerous changes in homeostasis parameters, leading to characteristic signs of pro-inflammatory stress, such as oxidative stress, inflammasome formation, and activation of pro-inflammatory cytokine signaling. This involvement of the JAK-STAT pathway in various physiological processes extends to neuromuscular excitation, cell proliferation and differentiation, homeostasis, embryogenesis, and the pathogenesis of various tumor diseases [69, 320].

The CNS, with its relatively stable homeostasis parameters and the presence of the blood–brain barrier, stands as a privileged tissue. Although normal human microglia exhibit low activity compared to other stromal macrophages, neurons in the CNS face unique challenges. Neurons rely on aerobic glucose oxidation, minimizing the risk of lipotoxicity and mitochondrial dysfunction, but they have limited regenerative potential and accumulate damage to the genome and proteome with aging, leading to pro-inflammatory stress, apoptosis, and a decrease in their numbers within the CNS [321, 322]. Due to the constant need for increased protein synthesis and substantial energy requirements, neurons are susceptible to proteinopathies, oxidative stress, and mitochondrial dysfunction. Furthermore, an unbalanced action of neurotransmitters can lead to excitotoxicity and neuronal damage, establishing a relationship between neurotransmission and cellular stress signaling pathways, including JAK-STAT [155, 180, 181].

Pro-inflammatory factors play a role in the normal functioning of the CNS, including neuronal communication, plasticity, learning, and memory [156, 321–323]. Dysfunctional pro-inflammatory mechanisms within the CNS can contribute to tissue maladaptation and the pathogenesis of various disorders, particularly depression and neurodegenerative diseases [155, 156, 322–324]. It should be noted that cellular stress mechanisms themselves can act as potential damaging factors, such as free radicals. As a result, cellular stress programs include negative feedback mechanisms to limit the severity, duration, and prevalence of stress. Understanding the underlying mechanisms of pro-inflammatory cellular and tissue stress is essential to understand both pathological and physiological processes, including psychoemotional stress.

The presented concept suggests the potential use of pharmacological modulators targeting inflammation and immunity in a wide range of diseases, including stress-associated pathologies.

## Conclusion and perspective

The role of the JAK-STAT signaling pathway in systemic homeostasis is multifaceted, encompassing not just classical inflammation, but also meta-inflammation and aging. These dimensions invite an examination of its relevance for stress-associated disorders. The involvement of the pathway in low-grade chronic inflammation provides an insightful framework to understand the reciprocal relationship between stress and systemic inflammation [38]. In addition, the complex interplay between stress and inflammation is underscored by the propensity of chronic stress to engender a pro-inflammatory state [317–319]. Such dynamics extend the imperative of studying the JAK-STAT pathway beyond isolated pathological conditions to a broader continuum involving stress and inflammation. Its role in functions of the central nervous system (CNS) such as neuronal communication, plasticity, learning, and memory accentuates its relevance [156, 321–325]. Emerging evidence suggests that Jakinibs can positively influence mental health outcomes in chronic conditions, including rheumatoid arthritis, thus expanding their potential applicability [267].

In the domain of targeted JAK inhibition, the advent of isoform-specific inhibitors such as filgotinib and upadacitinib signifies a new era of targeted pharmacology. These agents aim to enhance therapeutic efficacy while minimizing undesirable off-target effects, providing a sophisticated mechanism to modulate the JAK-STAT pathway [273–275]. This increase in specificity increases the suitability of Jakinibs for stress-associated disorders, where disruption of systemic homeostasis remains a notable concern.

In addition, the concept of combination therapies opens a new frontier in maximizing therapeutic specificity and potency. By coupling Jakinibs with agents such as monoclonal antibodies, there is the potential for synergistic effects that could amplify treatment outcomes [276]. This multimodal strategy is particularly relevant for the treatment of stress-related neuropsychiatric conditions, which often involve complex, multifactorial etiologies. The advent of precision drug delivery systems, including nanoparticle-based carriers such as liposomes, also promises to overcome the challenges posed by the blood–brain barrier, especially in the context of neuropsychiatric conditions [279].

In conclusion, the importance of the JAK-STAT pathway in stress-related disorders transcends academic speculation. The emergence of isoform-specific inhibitors, combination therapies, and precision drug delivery systems not only enriches our therapeutic arsenal but also adds nuance to our understanding of the role of the JAK-STAT pathway in the complex matrix of stress and inflammation. The extension of JAK-STAT inhibitors from their origins in classical immunology to their emerging role in stress-associated disorders marks a substantial shift, thus expanding both our theoretical understanding and the scope of potential therapeutic interventions.

### Supplementary Information

**Additional file 1: Supplementary Table S1.** List of JAK inhibitor’s studies, registered in Clinical Trials Registry (June 2023).

## Data Availability

Not applicable.

## References

[1] Ghoreschi K, Laurence A, O’Shea JJ (2009). Janus kinases in immune cell signaling. Immunol Rev.

[2] Seif F, Khoshmirsafa M, Aazami H, Mohsenzadegan M, Sedighi G, Bahar M (2017). The role of JAK-STAT signaling pathway and its regulators in the fate of T helper cells. Cell Commun Signal.

[3] Darnell JE, Kerr IM, Stark GR (1994). JAK-STAT pathways and transcriptional activation in response to IFNs and other extracellular signaling proteins. Science.

[4] Leonard WJ, O’Shea JJ (1998). Jaks and STATs: biological implications. Annu Rev Immunol..

[5] Beurel E, Toups M, Nemeroff CB (2020). The Bidirectional Relationship of Depression and Inflammation: Double Trouble. Neuron..

[6] Sarapultsev A, Sarapultsev P, Dremencov E, Komelkova M, Tseilikman O, Tseilikman V (2020). Low glucocorticoids in stress-related disorders: the role of inflammation. Stress.

[7] Almulla AF, Maes M (2023). Although serotonin is not a major player in depression, its precursor is. Mol Psychiatry..

[8] Tian F, Shen Q, Hu Y, Ye W, Valdimarsdóttir UA, Song H (2022). Association of stress-related disorders with subsequent risk of all-cause and cause-specific mortality: A population-based and sibling-controlled cohort study. Lancet Reg Health Euro.

[9] Troubat R, Barone P, Leman S, Desmidt T, Cressant A, Atanasova B (2021). Neuroinflammation and depression: A review. Eur J Neurosci.

[10] Goldstein DS (2023). Stress and the autonomic nervous system. Auton Neurosci.

[11] Lu S, Wei F, Li G (2021). The evolution of the concept of stress and the framework of the stress system. Cell Stress..

[12] Bienertova-Vasku J, Lenart P, Scheringer M (2020). Eustress and Distress: Neither Good Nor Bad, but Rather the Same?. BioEssays.

[13] Gusev EY, Zotova NV (2019). Cellular Stress and General Pathological Processes. Curr Pharm Des.

[14] Gusev E, Zhuravleva Y (2022). Inflammation: A New Look at an Old Problem. Int J Mol Sci.

[15] Brazhnikov A, Zotova N, Solomatina L, Sarapultsev A, Spirin A, Gusev E (2023). Shock-Associated Systemic Inflammation in Amniotic Fluid Embolism Complicated by Clinical Death. Pathophysiology.

[16] Zotova N, Zhuravleva Y, Chereshnev V, Gusev E (2023). Acute and Chronic Systemic Inflammation: Features and Differences in the Pathogenesis, and Integral Criteria for Verification and Differentiation. Int J Mol Sci.

[17] Gusev E, Sarapultsev A (2023). Atherosclerosis and Inflammation: Insights from the Theory of General Pathological Processes. Int J Mol Sci.

[18] Rajkumar RP (2023). Biomarkers of Neurodegeneration in Post-Traumatic Stress Disorder: An Integrative Review. Biomedicines.

[19] Qi G, Mi Y, Yin F (2020). Cellular Specificity and Intercellular Coordination in the Brain Bioenergetic System: Implications for Aging and Neurodegeneration. Front Physiol.

[20] Jelinek M, Jurajda M, Duris K (2021). Oxidative Stress in the Brain: Basic Concepts and Treatment Strategies in Stroke. Antioxidants (Basel).

[21] Cobley JN, Fiorello ML, Bailey DM (2018). 13 reasons why the brain is susceptible to oxidative stress. Redox Biol.

[22] Cooper JA, Nuutinen MR, Lawlor VM, DeVries BAM, Barrick EM, Hossein S (2021). Reduced adaptation of glutamatergic stress response is associated with pessimistic expectations in depression. Nat Commun.

[23] Dantzer R, Walker AK (2014). Is there a role for glutamate-mediated excitotoxicity in inflammation-induced depression?. J Neural Transm (Vienna).

[24] Wong TS, Li G, Li S, Gao W, Chen G, Gan S (2023). G protein-coupled receptors in neurodegenerative diseases and psychiatric disorders. Signal Transduct Target Ther.

[25] Yamanaka G, Hayashi K, Morishita N, Takeshita M, Ishii C, Suzuki S (2023). Experimental and Clinical Investigation of Cytokines in Migraine: A Narrative Review. Int J Mol Sci.

[26] Guzman-Martinez L, Maccioni RB, Andrade V, Navarrete LP, Pastor MG, Ramos-Escobar N (2019). Neuroinflammation as a Common Feature of Neurodegenerative Disorders. Front Pharmacol.

[27] Buckley PF (2019). Neuroinflammation and Schizophrenia. Curr Psychiatry Rep.

[28] Tanaka M, Toldi J, Vécsei L (2020). Exploring the Etiological Links behind Neurodegenerative Diseases: Inflammatory Cytokines and Bioactive Kynurenines. Int J Mol Sci.

[29] Johnson JD, Barnard DF, Kulp AC, Mehta DM (2019). Neuroendocrine Regulation of Brain Cytokines After Psychological Stress. J Endocr Soc.

[30] Gusev EY, Zhuravleva YA, Zotova NV (2019). Correlation of the evolution of immunity and inflammation in vertebrates. Biol Bull Rev.

[31] Liongue C, Ward AC (2013). Evolution of the JAK-STAT pathway. JAKSTAT.

[32] Liongue C, O'Sullivan LA, Trengove MC, Ward AC (2012). Evolution of JAK-STAT pathway components: mechanisms and role in immune system development. PLoS ONE.

[33] Gu Q, Kanungo J (2021). Effect of ketamine on gene expression in zebrafish embryos. J Appl Toxicol.

[34] Johnson DE, O'Keefe RA, Grandis JR (2018). Targeting the IL-6/JAK/STAT3 signaling axis in cancer. Nat Rev Clin Oncol.

[35] Hu X, Li J, Fu M, Zhao X, Wang W (2021). The JAK/STAT signaling pathway: from bench to clinic. Signal Transduct Target Ther.

[36] Gurzov EN, Stanley WJ, Pappas EG, Thomas HE, Gough DJ (2016). The JAK/STAT pathway in obesity and diabetes. FEBS J..

[37] Dodington DW, Desai HR, Woo M (2018). JAK/STAT - Emerging Players in Metabolism. Trends Endocrinol Metab.

[38] Ji Y, Li M, Chang M, Liu R, Qiu J, Wang K (2022). Inflammation: Roles in Skeletal Muscle Atrophy. Antioxidants (Basel).

[39] Milaneschi Y, Simmons WK, van Rossum EFC, Penninx BW (2019). Depression and obesity: evidence of shared biological mechanisms. Mol Psychiatry.

[40] Zhang M, Chen J, Yin Z, Wang L, Peng L (2021). The association between depression and metabolic syndrome and its components: a bidirectional two-sample Mendelian randomization study. Transl Psychiatry.

[41] Graham E, Deschênes SS, Rosella LC, Schmitz N (2021). Measures of depression and incident type 2 diabetes in a community sample. Ann Epidemiol.

[42] Jain M, Singh MK, Shyam H, Mishra A, Kumar S, Kumar A (2021). Role of JAK/STAT in the Neuroinflammation and its Association with Neurological Disorders. Ann Neurosci.

[43] Hu Q, Bian Q, Rong D, Wang L, Song J, Huang HS (2023). JAK/STAT pathway: Extracellular signals, diseases, immunity, and therapeutic regimens. Front Bioeng Biotechnol.

[44] Dudley AC, Thomas D, Best J, Jenkins A (2004). The STATs in cell stress-type responses. Cell Commun Signal.

[45] Al-Samhari MM, Al-Rasheed NM, Al-Rejaie S, Al-Rasheed NM, Hasan IH, Mahmoud AM (2016). Possible involvement of the JAK/STAT signaling pathway in N-acetylcysteine-mediated antidepressant-like effects. Exp Biol Med (Maywood).

[46] Nicolas CS, Amici M, Bortolotto ZA, Doherty A, Csaba Z, Fafouri A (2013). The role of JAK-STAT signaling within the CNS. JAK-STAT.

[47] Firmbach-Kraft I, Byers M, Shows T, Dalla-Favera R, Krolewski JJ (1990). Tyk2, prototype of a novel class of non-receptor tyrosine kinase genes. Oncogene.

[48] Pritchard MA, Baker E, Callen DF, Sutherland GR, Wilks AF (1992). Two members of the JAK family of protein tyrosine kinases map to chromosomes 1p31.3 and 9p24. Mamm Genome..

[49] Xin P, Xu X, Deng C, Liu S, Wang Y, Zhou X (2020). The role of JAK/STAT signaling pathway and its inhibitors in diseases. Int Immunopharmacol..

[50] Luo H, Rose P, Barber D, Hanratty WP, Lee S, Roberts TM, D’Andrea AD, Dearolf CR (1997). Mutation in the Jak kinase JH2 domain hyperactivates Drosophila and mammalian JAK-STAT pathways. Mol Cell Biol..

[51] Baines AJ (2006). A FERM-adjacent (FA) region defines a subset of the 4.1 superfamily and is a potential regulator of FERM domain function. BMC Genomics..

[52] Chen M, Cheng A, Chen YQ, Hymel A, Hanson EP, Kimmel L, Minami Y, Taniguchi T, Changelian PS, O’Shea JJ (1997). The amino terminus of JAK3 is necessary and sufficient for binding to the common gamma chain and confers the ability to transmit interleukin 2-mediated signals. Proc Natl Acad Sci USA.

[53] Shan Y, Gnanasambandan K, Ungureanu D, Kim ET, Hammarén H, Yamashita K, Silvennoinen O, Shaw DE, Hubbard SR (2014). Molecular basis for pseudokinase-dependent autoinhibition of JAK2 tyrosine kinase. Nat Struct Mol Biol.

[54] Argetsinger LS, Kouadio J-LK, Steen H, Stensballe A, Jensen ON, Carter-Su C (2004). Autophosphorylation of JAK2 on tyrosines 221 and 570 regulates its activity. Mol Cell Biol..

[55] Banerjee S, Biehl A, Gadina M, Hasni S, Schwartz DM (2017). JAK-STAT Signaling as a Target for Inflammatory and Autoimmune Diseases: Current and Future Prospects. Drugs.

[56] Stofega MR, Herrington J, Billestrup N, Carter-Su C (2000). Mutation of the SHP-2 binding site in growth hormone (GH) receptor prolongs GH-promoted tyrosyl phosphorylation of GH receptor, JAK2, and STAT5B. Mol Endocrinol.

[57] Hibi M, Murakami M, Saito M, Hirano T, Taga T, Kishimoto T (1990). Molecular cloning and expression of an IL-6 signal transducer, gp130. Cell..

[58] Wilks AF (2008). The JAK kinases: not just another kinase drug discovery target. Semin Cell Dev Biol.

[59] Jatiani SS, Baker SJ, Silverman LR, Reddy EP (2010). Jak/STAT pathways in cytokine signaling and myeloproliferative disorders: approaches for targeted therapies. Genes Cancer..

[60] Schindler C, Levy DE, Decker T (2007). JAK-STAT signaling: from interferons to cytokines. The J Biol Chem.

[61] Lu Y, Zhou J, Xu C, Lin H, Xiao J, Wang Z, Yang B (2008). JAK/STAT and PI3K/AKT pathways form a mutual transactivation loop and afford resistance to oxidative stress-induced apoptosis in cardiomyocytes. Cell Physiol Biochem.

[62] Zegeye MM, Lindkvist M, Fälker K, Kumawat AK, Paramel G, Grenegård M (2018). Activation of the JAK/STAT3 and PI3K/AKT pathways are crucial for IL-6 trans-signaling-mediated pro-inflammatory response in human vascular endothelial cells. Cell Commun Signal.

[63] Morris R, Kershaw NJ, Babon JJ (2018). The molecular details of cytokine signaling via the JAK/STAT pathway. Protein Sci..

[64] Agashe RP, Lippman SM, Kurzrock R (2022). JAK: Not Just Another Kinase. Mol Cancer Ther.

[65] Bousoik E, Montazeri Aliabadi H (2018). “Do We Know Jack” About JAK? A Closer Look at JAK/STAT Signaling Pathway. Front Oncol..

[66] Babon JJ, Lucet IS, Murphy JM, Nicola NA, Varghese LN (2014). The Molecular Regulation of Janus Kinase (jak) Activation. Biochem J.

[67] Yoshimura A, Naka T, Kubo M (2007). SOCS proteins, cytokine signalling and immune regulation. Nat Rev Immunol.

[68] Yoshimura A, Ito M, Mise-Omata S, Ando M (2021). SOCS: negative regulators of cytokine signaling for immune tolerance. Int Immunol..

[69] Tamiya T, Kashiwagi I, Takahashi R, Yasukawa H, Yoshimura A (2011). Suppressors of cytokine signaling (SOCS) proteins and JAK/STAT pathways: regulation of T-cell inflammation by SOCS1 and SOCS3. Arterioscler Thromb Vasc Biol..

[70] Xu D, Qu C-K (2008). Protein tyrosine phosphatases in the JAK/STAT pathway. Front Biosci.

[71] Castelo-Soccio L, Kim H, Gadina M, Schwartzberg PL, Laurence A, O’Shea JJ. Protein kinases: drug targets for immunological disorders. Nat Rev Immunol. 2023;1–20. 10.1038/s41577-023-00877-7. 10.1038/s41577-023-00877-7 PMC10184645 37188939

[72] Zahn M, Kaluszniak B, Möller P, Marienfeld R (2021). The PTP1B mutant PTP1B∆2-4 is a positive regulator of the JAK/STAT signalling pathway in Hodgkin lymphoma. Carcinogenesis.

[73] Myers MP, Andersen JN, Cheng A, Tremblay ML, Horvath CM, Parisien JP, Salmeen A, Barford D, Tonks NK (2001). TYK2 and JAK2 are substrates of protein-tyrosine phosphatase 1B. J Biol Chem.

[74] Shuai K, Liu B (2003). Regulation of JAK-STAT signalling in the immune system. Nat Rev Immunol.

[75] Kim H, Hawley TS, Hawley RG, Baumann H (1998). Protein tyrosine phosphatase 2 (SHP-2) moderates signaling by gp130 but is not required for the induction of acute-phase plasma protein genes in hepatic cells. Mol Cell Biol.

[76] Al Barashdi MA, Ali A, McMullin MF, Mills K (2021). Protein tyrosine phosphatase receptor type C (PTPRC or CD45). J Clin Pathol.

[77] Saunders AE, Johnson P (2010). Modulation of immune cell signalling by the leukocyte common tyrosine phosphatase, CD45. Cell Signal.

[78] Tchilian EZ, Beverley PCL (2006). Altered CD45 expression and disease. Trends Immunol.

[79] Niu G-J, Xu J-D, Yuan W-J, Sun J-J, Yang M-C, He Z-H (2018). Protein Inhibitor of Activated STAT (PIAS) Negatively Regulates the JAK/STAT Pathway by Inhibiting STAT Phosphorylation and Translocation. Front Immunol.

[80] Velazquez L (2012). The Lnk adaptor protein: a key regulator of normal and pathological hematopoiesis. Arch Immunol Ther Exp (Warsz)..

[81] Stahl N, Boulton TG, Farruggella T, Ip NY, Davis S, Witthuhn BA, Quelle FW, Silvennoinen O, Barbieri G, Pellegrini S (1994). Association and activation of Jak-Tyk kinases by CNTF-LIF-OSM-IL-6 beta receptor components. Science.

[82] Tesoriere A, Dinarello A, Argenton F (2021). The Roles of Post-Translational Modifications in STAT3 Biological Activities and Functions. Biomedicines.

[83] Saharinen P, Vihinen M, Silvennoinen O (2003). Autoinhibition of Jak2 tyrosine kinase is dependent on specific regions in its pseudokinase domain. Mol Biol Cell.

[84] Saharinen P, Takaluoma K, Silvennoinen O (2000). Regulation of the Jak2 tyrosine kinase by its pseudokinase domain. Mol Cell Biol.

[85] Ding N, Miller SA, Savant SS, O’Hagan HM (2019). JAK2 regulates mismatch repair protein-mediated epigenetic alterations in response to oxidative damage. Environ Mol Mutagen.

[86] Rui L, Drennan AC, Ceribelli M, Zhu F, Wright GW, Huang DW (2016). Epigenetic gene regulation by Janus kinase 1 in diffuse large B-cell lymphoma. Proc Natl Acad Sci USA.

[87] Vadivel CK, Gluud M, Torres-Rusillo S, Boding L, Willerslev-Olsen A, Buus TB (2021). JAK3 Is Expressed in the Nucleus of Malignant T Cells in Cutaneous T Cell Lymphoma (CTCL). Cancers (Basel).

[88] O’Shea JJ, Plenge R (2012). JAK and STAT signaling molecules in immunoregulation and immune-mediated disease. Immunity.

[89] Suzuki K, Nakajima H, Saito Y, Saito T, Leonard WJ, Iwamoto I (2000). Janus kinase 3 (Jak3) is essential for common cytokine receptor gamma chain (gamma(c))-dependent signaling: comparative analysis of gamma(c), Jak3, and gamma(c) and Jak3 double-deficient mice. Int Immunol.

[90] Gusev E, Sarapultsev A, Hu D, Chereshnev V (2021). Problems of Pathogenesis and Pathogenetic Therapy of COVID-19 from the Perspective of the General Theory of Pathological Systems (General Pathological Processes). Int J Mol Sci.

[91] Frede N, Lorenzetti R, Hüppe JM, Janowska I, Troilo A, Schleyer M-T (2023). JAK inhibitors differentially modulate B cell activation, maturation and function: A comparative analysis of five JAK inhibitors in an in-vitro B cell differentiation model and in patients with rheumatoid arthritis. Front Immunol.

[92] Imada K, Bloom ET, Nakajima H, Horvath-Arcidiacono JA, Udy GB, Davey HW (1998). Stat5b is essential for natural killer cell-mediated proliferation and cytolytic activity. J Exp Med.

[93] Gotthardt D, Trifinopoulos J, Sexl V, Putz EM (2019). JAK/STAT Cytokine Signaling at the Crossroad of NK Cell Development and Maturation. Front Immunol.

[94] Oku S, Takenaka K, Kuriyama T, Shide K, Kumano T, Kikushige Y (2010). JAK2 V617F uses distinct signalling pathways to induce cell proliferation and neutrophil activation. Br J Haematol.

[95] Rauprich P, Kasper B, Tidow N, Welte K (1995). The protein tyrosine kinase JAK2 is activated in neutrophils from patients with severe congenital neutropenia. Blood.

[96] Yan Z, Gibson SA, Buckley JA, Qin H, Benveniste EN (2018). Role of the JAK/STAT signaling pathway in regulation of innate immunity in neuroinflammatory diseases. Clin Immunol.

[97] Yoshida S, Yamada S, Yokose K, Matsumoto H, Fujita Y, Asano T (2021). Interferon-γ induces interleukin-6 production by neutrophils via the Janus kinase (JAK)-signal transducer and activator of transcription (STAT) pathway. BMC Res Notes.

[98] Bhattacharjee A, Pal S, Feldman GM, Cathcart MK (2011). Hck is a key regulator of gene expression in alternatively activated human monocytes. J Biol Chem.

[99] Roy B, Bhattacharjee A, Xu B, Ford D, Maizel AL, Cathcart MK (2002). IL-13 signal transduction in human monocytes: phosphorylation of receptor components, association with Jaks, and phosphorylation/activation of Stats. J Leukoc Biol.

[100] Musso T, Johnston JA, Linnekin D, Varesio L, Rowe TK, O’Shea JJ (1995). Regulation of JAK3 expression in human monocytes: phosphorylation in response to interleukins 2, 4, and 7. J Exp Med.

[101] Vogel A, Martin K, Soukup K, Halfmann A, Kerndl M, Brunner JS (2022). JAK1 signaling in dendritic cells promotes peripheral tolerance in autoimmunity through PD-L1-mediated regulatory T cell induction. Cell Rep.

[102] Klaeschen AS, Nümm TJ, Herrmann N, Leib N, Maintz L, Sakai T (2021). JAK1/2 inhibition impairs the development and function of inflammatory dendritic epidermal cells in atopic dermatitis. J Allergy Clin Immunol.

[103] Spinelli FR, Colbert RA, Gadina M (2021). JAK1: Number one in the family; number one in inflammation?. Rheumatology (Oxford)..

[104] Rodig SJ, Meraz MA, White JM, Lampe PA, Riley JK, Arthur CD (1998). Disruption of the Jak1 gene demonstrates obligatory and nonredundant roles of the Jaks in cytokine-induced biologic responses. Cell.

[105] Kleppe M, Spitzer MH, Li S, Hill CE, Dong L, Papalexi E (2017). Jak1 Integrates Cytokine Sensing to Regulate Hematopoietic Stem Cell Function and Stress Hematopoiesis. Cell Stem Cell.

[106] Ren J, Kolli D, Liu T, Xu R, Garofalo RP, Casola A (2011). Human metapneumovirus inhibits IFN-β signaling by downregulating Jak1 and Tyk2 cellular levels. PLoS ONE.

[107] Miller DM, Rahill BM, Boss JM, Lairmore MD, Durbin JE, Waldman JW (1998). Human cytomegalovirus inhibits major histocompatibility complex class II expression by disruption of the Jak/Stat pathway. J Exp Med.

[108] Wu Y, Liu Q, Zhou J, Xie W, Chen C, Wang Z (2017). Zika virus evades interferon-mediated antiviral response through the co-operation of multiple nonstructural proteins in vitro. Cell Discov.

[109] Tuttle KD, Waugh KA, Araya P, Minter R, Orlicky DJ, Ludwig M (2020). JAK1 Inhibition Blocks Lethal Immune Hypersensitivity in a Mouse Model of Down Syndrome. Cell Rep.

[110] Ren Y, Zhang Y, Liu RZ, Fenstermacher DA, Wright KL, Teer JK (2013). JAK1 truncating mutations in gynecologic cancer define new role of cancer-associated protein tyrosine kinase aberrations. Sci Rep.

[111] Albacker LA, Wu J, Smith P, Warmuth M, Stephens PJ, Zhu P (2017). Loss of function JAK1 mutations occur at high frequency in cancers with microsatellite instability and are suggestive of immune evasion. PLoS ONE.

[112] Han P, Dai Q, Fan L, Lin H, Zhang X, Li F (2019). Genome-Wide CRISPR Screening Identifies JAK1 Deficiency as a Mechanism of T-Cell Resistance. Front Immunol.

[113] Witalisz-Siepracka A, Klein K, Prinz D, Leidenfrost N, Schabbauer G, Dohnal A (2018). Loss of JAK1 Drives Innate Immune Deficiency. Front Immunol.

[114] Daza-Cajigal V, Albuquerque AS, Pearson J, Hinley J, Mason AS, Stahlschmidt J (2019). Loss of Janus Associated Kinase 1 Alters Urothelial Cell Function and Facilitates the Development of Bladder Cancer. Front Immunol.

[115] Ihle JN, Witthuhn BA, Quelle FW, Yamamoto K, Silvennoinen O (1995). Signaling through the hematopoietic cytokine receptors. Annu Rev Immunol.

[116] Vargas-Hernández A, Forbes LR (2019). JAK/STAT proteins and their biological impact on NK cell development and function. Mol Immunol.

[117] Cao Y, Wang J, Jiang S, Lyu M, Zhao F, Liu J (2023). JAK1/2 inhibitor ruxolitinib promotes the expansion and suppressive action of polymorphonuclear myeloid-derived suppressor cells via the JAK/STAT and ROS-MAPK/NF-κB signalling pathways in acute graft-versus-host disease. Clin Transl Immunology.

[118] Biggs CM, Cordeiro-Santanach A, Prykhozhij SV, Deveau AP, Lin Y, Del Bel KL (2022). Human JAK1 gain of function causes dysregulated myelopoeisis and severe allergic inflammation. JCI insight.

[119] Waters MJ, Brooks AJ (2015). JAK2 activation by growth hormone and other cytokines. Biochem J.

[120] Ihle JN (1995). Cytokine receptor signalling. Nature.

[121] Parganas E, Wang D, Stravopodis D, Topham DJ, Marine JC, Teglund S (1998). Jak2 is essential for signaling through a variety of cytokine receptors. Cell.

[122] Pelletier S, Gingras S, Funakoshi-Tago M, Howell S, Ihle JN (2006). Two domains of the erythropoietin receptor are sufficient for Jak2 binding/activation and function. Mol Cell Biol.

[123] Ingley E (2012). Integrating novel signaling pathways involved in erythropoiesis. IUBMB Life.

[124] Besancenot R, Roos-Weil D, Tonetti C, Abdelouahab H, Lacout C, Pasquier F (2014). JAK2 and MPL protein levels determine TPO-induced megakaryocyte proliferation vs differentiation. Blood..

[125] Tian SS, Tapley P, Sincich C, Stein RB, Rosen J, Lamb P (1996). Multiple signaling pathways induced by granulocyte colony-stimulating factor involving activation of JAKs, STAT5, and/or STAT3 are required for regulation of three distinct classes of immediate early genes. Blood.

[126] Lehtonen A, Matikainen S, Miettinen M, Julkunen I (2002). Granulocyte-macrophage colony-stimulating factor (GM-CSF)-induced STAT5 activation and target-gene expression during human monocyte/macrophage differentiation. J Leukoc Biol.

[127] Wang L, Xue J, Zadorozny EV, Robinson LJ (2008). G-CSF stimulates Jak2-dependent Gab2 phosphorylation leading to Erk1/2 activation and cell proliferation. Cell Signal.

[128] Morgan KJ, Gilliland DG (2008). A role for JAK2 mutations in myeloproliferative diseases. Annu Rev Med.

[129] Akada H, Akada S, Hutchison RE, Sakamoto K, Wagner K-U, Mohi G (2014). Critical role of Jak2 in the maintenance and function of adult hematopoietic stem cells. Stem Cells.

[130] Waickman AT, Park J-Y, Park J-H (2016). The common γ-chain cytokine receptor: tricks-and-treats for T cells. Cell Mol Life Sci.

[131] Hercus TR, Thomas D, Guthridge MA, Ekert PG, King-Scott J, Parker MW (2009). The granulocyte-macrophage colony-stimulating factor receptor: linking its structure to cell signaling and its role in disease. Blood.

[132] Wang H, Brown J, Gao S, Liang S, Jotwani R, Zhou H (2013). The role of JAK-3 in regulating TLR-mediated inflammatory cytokine production in innate immune cells. J Immunol.

[133] Park SY, Saijo K, Takahashi T, Osawa M, Arase H, Hirayama N (1995). Developmental defects of lymphoid cells in Jak3 kinase-deficient mice. Immunity.

[134] Rane SG, Mangan JK, Amanullah A, Wong BC, Vora RK, Liebermann DA (2002). Activation of the Jak3 pathway is associated with granulocytic differentiation of myeloid precursor cells. Blood.

[135] Bhavsar SK, Gu S, Bobbala D, Lang F (2011). Janus kinase 3 is expressed in erythrocytes, phosphorylated upon energy depletion and involved in the regulation of suicidal erythrocyte death. Cell Physiol Biochem.

[136] Alghareeb SA, Alfhili MA, Fatima S (2023). Molecular Mechanisms and Pathophysiological Significance of Eryptosis. Int J Mol Sci.

[137] Verbsky JW, Bach EA, Fang YF, Yang L, Randolph DA, Fields LE (1996). Expression of Janus kinase 3 in human endothelial and other non-lymphoid and non-myeloid cells. J Biol Chem.

[138] Barcia Durán JG, Lu T, Houghton S, Geng F, Schreiner R, Xiang J (2021). Endothelial Jak3 expression enhances pro-hematopoietic angiocrine function in mice. Commun Biol.

[139] Butler JM, Nolan DJ, Vertes EL, Varnum-Finney B, Kobayashi H, Hooper AT (2010). Endothelial cells are essential for the self-renewal and repopulation of Notch-dependent hematopoietic stem cells. Cell Stem Cell.

[140] Derecka M, Gornicka A, Koralov SB, Szczepanek K, Morgan M, Raje V (2012). Tyk2 and Stat3 regulate brown adipose tissue differentiation and obesity. Cell Metab.

[141] Ricardo-Gonzalez RR, Red Eagle A, Odegaard JI, Jouihan H, Morel CR, Heredia JE (2010). IL-4/STAT6 immune axis regulates peripheral nutrient metabolism and insulin sensitivity. Proc Natl Acad Sci USA.

[142] Dobrian AD, Galkina EV, Ma Q, Hatcher M, Aye SM, Butcher MJ (2013). STAT4 deficiency reduces obesity-induced insulin resistance and adipose tissue inflammation. Diabetes.

[143] Muromoto R, Oritani K, Matsuda T (2022). Current understanding of the role of tyrosine kinase 2 signaling in immune responses. World J Biol Chem.

[144] Muromoto R, Shimoda K, Oritani K, Matsuda T (2021). Therapeutic Advantage of Tyk2 Inhibition for Treating Autoimmune and Chronic Inflammatory Diseases. Biol Pharm Bull.

[145] Shimoda HK, Shide K, Kameda T, Matsunaga T, Shimoda K (2010). Tyrosine kinase 2 interacts with the proapoptotic protein Siva-1 and augments its apoptotic functions. Biochem Biophys Res Commun.

[146] Simonović N, Witalisz-Siepracka A, Meissl K, Lassnig C, Reichart U, Kolbe T (2019). NK Cells Require Cell-Extrinsic and -Intrinsic TYK2 for Full Functionality in Tumor Surveillance and Antibacterial Immunity. J Immunol.

[147] Li F, Zhang R, Hu C, Ran Q, Xiang Y, Xiang L (2021). Irradiation Haematopoiesis Recovery Orchestrated by IL-12/IL-12Rβ1/TYK2/STAT3-Initiated Osteogenic Differentiation of Mouse Bone Marrow-Derived Mesenchymal Stem Cells. Front Cell Dev Biol.

[148] Tokumasa N, Suto A, Kagami S-I, Furuta S, Hirose K, Watanabe N (2007). Expression of Tyk2 in dendritic cells is required for IL-12, IL-23, and IFN-gamma production and the induction of Th1 cell differentiation. Blood.

[149] Fan C-S, Chen C-C, Chen L-L, Chua KV, Hung H-C, Hsu JT-A (2022). Extracellular HSP90α Induces MyD88-IRAK Complex-Associated IKKα/β-NF-κB/IRF3 and JAK2/TYK2-STAT-3 Signaling in Macrophages for Tumor-Promoting M2-Polarization. Cells..

[150] Poelzl A, Lassnig C, Tangermann S, Hromadová D, Reichart U, Gawish R (2021). TYK2 licenses non-canonical inflammasome activation during endotoxemia. Cell Death Differ.

[151] Hirashima K, Muromoto R, Minoguchi H, Matsumoto T, Kitai Y, Kashiwakura J-I (2020). The mechanism of Tyk2 deficiency-induced immunosuppression in mice involves robust IL-10 production in macrophages. Cytokine.

[152] Chung BM, Kang HC, Han SY, Heo HS, Lee JJ, Jeon J (2006). Jak2 and Tyk2 are necessary for lineage-specific differentiation, but not for the maintenance of self-renewal of mouse embryonic stem cells. Biochem Biophys Res Commun.

[153] Bellucci S, Michiels JJ (2006). The role of JAK2 V617F mutation, spontaneous erythropoiesis and megakaryocytopoiesis, hypersensitive platelets, activated leukocytes, and endothelial cells in the etiology of thrombotic manifestations in polycythemia vera and essential thrombocythemia. Semin Thromb Hemost.

[154] Mishra J, Verma RK, Alpini G, Meng F, Kumar N (2015). Role of Janus Kinase 3 in Predisposition to Obesity-associated Metabolic Syndrome. J Biol Chem.

[155] De-Fraja C, Conti L, Magrassi L, Govoni S, Cattaneo E (1998). Members of the JAK/STAT proteins are expressed and regulated during development in the mammalian forebrain. J Neurosci Res.

[156] Nicolas CS, Peineau S, Amici M, Csaba Z, Fafouri A, Javalet C (2012). The Jak/STAT pathway is involved in synaptic plasticity. Neuron.

[157] He F, Ge W, Martinowich K, Becker-Catania S, Coskun V, Zhu W (2005). A positive autoregulatory loop of JAK-STAT signaling controls the onset of astrogliogenesis. Nat Neurosci.

[158] Bonni A, Sun Y, Nadal-Vicens M, Bhatt A, Frank DA, Rozovsky I (1997). Regulation of gliogenesis in the central nervous system by the JAK-STAT signaling pathway. Science.

[159] Garza JC, Guo M, Zhang W, Lu X-Y (2008). Leptin increases adult hippocampal neurogenesis in vivo and in vitro. J Biol Chem.

[160] Kim YH, Chung J-I, Woo HG, Jung Y-S, Lee SH, Moon C-H (2010). Differential regulation of proliferation and differentiation in neural precursor cells by the Jak pathway. Stem Cells.

[161] Moult PR, Harvey J (2008). Hormonal regulation of hippocampal dendritic morphology and synaptic plasticity. Cell Adh Migr.

[162] Singh RK, Jia C, Garcia F, Carrasco GA, Battaglia G, Muma NA (2010). Activation of the JAK-STAT pathway by olanzapine is necessary for desensitization of serotonin2A receptor-stimulated phospholipase C signaling in rat frontal cortex but not serotonin2A receptor-stimulated hormone release. J Psychopharmacol (Oxford).

[163] Gray JA, Roth BL (2001). Paradoxical trafficking and regulation of 5-HT(2A) receptors by agonists and antagonists. Brain Res Bull.

[164] Guillet-Deniau I, Burnol AF, Girard J (1997). Identification and localization of a skeletal muscle secrotonin 5-HT2A receptor coupled to the Jak/STAT pathway. J Biol Chem.

[165] Muma NA, Singh RK, Vercillo MS, D’Souza DN, Zemaitaitis B, Garcia F (2007). Chronic olanzapine activates the Stat3 signal transduction pathway and alters expression of components of the 5-HT2A receptor signaling system in rat frontal cortex. Neuropharmacology.

[166] Oufkir T, Arseneault M, Sanderson JT, Vaillancourt C (2010). The 5-HT 2A serotonin receptor enhances cell viability, affects cell cycle progression and activates MEK-ERK1/2 and JAK2-STAT3 signalling pathways in human choriocarcinoma cell lines. Placenta.

[167] Oufkir T, Vaillancourt C (2011). Phosphorylation of JAK2 by serotonin 5-HT (2A) receptor activates both STAT3 and ERK1/2 pathways and increases growth of JEG-3 human placental choriocarcinoma cell. Placenta.

[168] Banes AKL, Shaw SM, Tawfik A, Patel BP, Ogbi S, Fulton D (2005). Activation of the JAK/STAT pathway in vascular smooth muscle by serotonin. Am J Physiol Cell Physiol.

[169] Rawlings JS, Rosler KM, Harrison DA (2004). The JAK/STAT signaling pathway. J Cell Sci.

[170] Singh RK, Shi J, Zemaitaitis BW, Muma NA (2007). Olanzapine increases RGS7 protein expression via stimulation of the Janus tyrosine kinase-signal transducer and activator of transcription signaling cascade. J Pharmacol Exp Ther.

[171] Mojiri-Forushani H, Khajehali E, Adelipour M, Mohammadi A (2023). Inhibitory effects of fluoxetine on the secretion of inflammatory mediators and JAK/STAT3 and JNK/TLR4 gene expression. Mol Biol Rep.

[172] Kabiri M, Hemmatpour A, Zare F, Hadinedoushan H, Karimollah A (2020). Paroxetine modulates immune responses by activating a JAK2/STAT3 signaling pathway. J Biochem Mol Toxicol.

[173] Mihara M, Hashizume M, Yoshida H, Suzuki M, Shiina M (2012). IL-6/IL-6 receptor system and its role in physiological and pathological conditions. Clinical Science (London, England 1979)..

[174] Rose-John S, Jenkins BJ, Garbers C, Moll JM, Scheller J (2023). Targeting IL-6 trans-signalling: past, present and future prospects. Nat Rev Immunol.

[175] Maes M, Anderson G, Kubera M, Berk M. Targeting classical IL-6 signalling or IL-6 trans-signalling in depression? Expert Opin Ther Targets. 2014;18(5). 10.1517/14728222.2014.888417. 10.1517/14728222.2014.888417 24548241

[176] Reisinger S. Transcriptional and behavioural modulation by serotonergic STAT3 relevant to mood and psychotic disorders. 2020. http://repositorium.meduniwien.ac.at/obvumwhs/5698012. Accessed 2023 Jun 27.

[177] Kong E, Sucic S, Monje FJ, Reisinger SN, Savalli G, Diao W (2015). STAT3 controls IL6-dependent regulation of serotonin transporter function and depression-like behavior. Sci Rep.

[178] Beurel E, Jope RS (2008). Differential regulation of STAT family members by glycogen synthase kinase-3. J Biol Chem.

[179] Minashima T, Zhang Y, Lee Y, Kirsch T (2013). Lithium chloride - a novel treatment for osteoarthritis?. Osteoarthr Cartil.

[180] Reisinger SN, Sideromenos S, Horvath O, Derdak S, Cicvaric A, Monje FJ, Bilban M, Häring M, Glat M, Pollak DD (2021). STAT3 in the dorsal raphe gates behavioural reactivity and regulates gene networks associated with psychopathology. Mol Psychiatry.

[181] Yu XM, Salter MW (1999). Src, a molecular switch governing gain control of synaptic transmission mediated by N-methyl-D-aspartate receptors. Proc Natl Acad Sci USA.

[182] Orellana DI, Quintanilla RA, Gonzalez-Billault C, Maccioni RB (2005). Role of the JAKs/STATs pathway in the intracellular calcium changes induced by interleukin-6 in hippocampal neurons. Neurotox Res.

[183] Chiba T, Yamada M, Aiso S (2009). Targeting the JAK2/STAT3 axis in Alzheimer’s disease. Expert Opin Ther Targets.

[184] Chiba T, Yamada M, Sasabe J, Terashita K, Shimoda M, Matsuoka M (2009). Amyloid-beta causes memory impairment by disturbing the JAK2/STAT3 axis in hippocampal neurons. Mol Psychiatry.

[185] Liu Y, Gibson SA, Benveniste EN, Qin H (2015). Opportunities for Translation from the Bench: Therapeutic Intervention of the JAK/STAT Pathway in Neuroinflammatory Diseases. Crit Rev Immunol.

[186] Regis G, Pensa S, Boselli D, Novelli F, Poli V (2008). Ups and downs: the STAT1:STAT3 seesaw of Interferon and gp130 receptor signalling. Semin Cell Dev Biol.

[187] Qin H, Buckley JA, Li X, Liu Y, Fox TH, Meares GP (2016). Inhibition of the JAK/STAT Pathway Protects Against α-Synuclein-Induced Neuroinflammation and Dopaminergic Neurodegeneration. J Neurosci.

[188] Nabavi SM, Ahmed T, Nawaz M, Devi KP, Balan DJ, Pittalà V (2019). Targeting STATs in neuroinflammation: The road less traveled!. Pharmacol Res.

[189] Butturini E, Boriero D, Carcereri de Prati A, Mariotto S (2019). STAT1 drives M1 microglia activation and neuroinflammation under hypoxia. Arch Biochem Biophys..

[190] Turnquist C, Wang Y, Severson DT, Zhong S, Sun B, Ma J (2014). STAT1-induced ASPP2 transcription identifies a link between neuroinflammation, cell polarity, and tumor suppression. Proc Natl Acad Sci USA.

[191] Liu N, Zhou Q, Wang H, Li Q, Chen Z, Lin Y (2023). MiRNA-338-3p Inhibits Neuroinflammation in the Corpus Callosum of LCV-LPS Rats Via STAT1 Signal Pathway. Cell Mol Neurobiol.

[192] Zang C-X, Wang L, Yang H-Y, Shang J-M, Liu H, Zhang Z-H (2022). HACE1 negatively regulates neuroinflammation through ubiquitylating and degrading Rac1 in Parkinson’s disease models. Acta Pharmacol Sin.

[193] Li T, Li L, Peng R, Hao H, Zhang H, Gao Y (2022). Abrocitinib Attenuates Microglia-Mediated Neuroinflammation after Traumatic Brain Injury via Inhibiting the JAK1/STAT1/NF-κB Pathway. Cells.

[194] Wang F, Xia J-J, Shen L-J, Jiang T-T, Li W-L, You D-L (2022). Curcumin attenuates intracerebral hemorrhage-induced neuronal apoptosis and neuroinflammation by suppressing JAK1/STAT1 pathway. Biochem Cell Biol.

[195] Khorooshi R, Babcock AA, Owens T (2008). NF-kappaB-driven STAT2 and CCL2 expression in astrocytes in response to brain injury. J Immunol.

[196] Rauch I, Müller M, Decker T (2013). The regulation of inflammation by interferons and their STATs. JAK-STAT.

[197] Zheng ZV, Chen J, Lyu H, Lam SYE, Lu G, Chan WY (2022). Novel role of STAT3 in microglia-dependent neuroinflammation after experimental subarachnoid haemorrhage. Stroke Vasc Neurol.

[198] Millot P, San C, Bennana E, Porte B, Vignal N, Hugon J (2020). STAT3 inhibition protects against neuroinflammation and BACE1 upregulation induced by systemic inflammation. Immunol Lett.

[199] Hu Y, Zhang X, Zhang J, Xia X, Li H, Qiu C (2021). Activated STAT3 signaling pathway by ligature-induced periodontitis could contribute to neuroinflammation and cognitive impairment in rats. J Neuroinflammation.

[200] Ahmad SF, Nadeem A, Ansari MA, Bakheet SA, Shahid M, Al-Mazroua HA (2022). CC chemokine receptor 5 antagonist alleviates inflammation by regulating IFN-γ/IL-10 and STAT4/Smad3 signaling in a mouse model of autoimmune encephalomyelitis. Cell Immunol.

[201] Natarajan C, Sriram S, Muthian G, Bright JJ (2004). Signaling through JAK2-STAT5 pathway is essential for IL-3-induced activation of microglia. Glia.

[202] Pu Z, Xia S, Shao P, Bao X, Wu D, Xu Y (2022). Regulation of Microglia-Activation-Mediated Neuroinflammation to Ameliorate Ischemia-Reperfusion Injury via the STAT5-NF-κB Pathway in Ischemic Stroke. Brain Sci.

[203] Monaghan KL, Aesoph D, Ammer AG, Zheng W, Rahimpour S, Farris BY (2021). Tetramerization of STAT5 promotes autoimmune-mediated neuroinflammation. Proc Natl Acad Sci USA.

[204] Sheng W, Yang F, Zhou Y, Yang H, Low PY, Kemeny DM (2014). STAT5 programs a distinct subset of GM-CSF-producing T helper cells that is essential for autoimmune neuroinflammation. Cell Res.

[205] Lawson BR, Gonzalez-Quintial R, Eleftheriadis T, Farrar MA, Miller SD, Sauer K (2015). Interleukin-7 is required for CD4(+) T cell activation and autoimmune neuroinflammation. Clin Immunol.

[206] He Y, Gao Y, Zhang Q, Zhou G, Cao F, Yao S (2020). IL-4 Switches Microglia/macrophage M1/M2 Polarization and Alleviates Neurological Damage by Modulating the JAK1/STAT6 Pathway Following ICH. Neuroscience.

[207] Xie L, Liu Y, Zhang N, Li C, Sandhu AF, Williams G (2021). Electroacupuncture Improves M2 Microglia Polarization and Glia Anti-inflammation of Hippocampus in Alzheimer’s Disease. Front Neurosci.

[208] Yao G, Bai Z, Niu J, Zhang R, Lu Y, Gao T, Wang H (2022). Astragalin attenuates depression-like behaviors and memory deficits and promotes M2 microglia polarization by regulating IL-4R/JAK1/STAT6 signaling pathway in a murine model of perimenopausal depression. Psychopharmacology.

[209] Im JH, Yeo IJ, Park PH, Choi DY, Han S-B, Yun J (2020). Deletion of Chitinase-3-like 1 accelerates stroke development through enhancement of Neuroinflammation by STAT6-dependent M2 microglial inactivation in Chitinase-3-like 1 knockout mice. Exp Neurol.

[210] Lashgari N-A, Roudsari NM, Momtaz S, Sathyapalan T, Abdolghaffari AH, Sahebkar A (2021). The involvement of JAK/STAT signaling pathway in the treatment of Parkinson’s disease. J Neuroimmunol.

[211] O’Shea JJ, Schwartz DM, Villarino AV, Gadina M, McInnes IB, Laurence A (2015). The JAK-STAT pathway: impact on human disease and therapeutic intervention. Annu Rev Med.

[212] Hamilton JA (2008). Colony-stimulating factors in inflammation and autoimmunity. Nat Rev Immunol.

[213] Meares GP, Liu Y, Rajbhandari R, Qin H, Nozell SE, Mobley JA (2014). PERK-dependent activation of JAK1 and STAT3 contributes to endoplasmic reticulum stress-induced inflammation. Mol Cell Biol.

[214] Rusek M, Smith J, El-Khatib K, Aikins K, Czuczwar SJ, Pluta R (2023). The Role of the JAK/STAT Signaling Pathway in the Pathogenesis of Alzheimer’s Disease: New Potential Treatment Target. Int J Mol Sci.

[215] Ren X, Zou L, Zhang X, Branco V, Wang J, Carvalho C (2017). Redox Signaling Mediated by Thioredoxin and Glutathione Systems in the Central Nervous System. Antioxid Redox Signal.

[216] Kaur N, Lu B, Monroe RK, Ward SM, Halvorsen SW (2005). Inducers of oxidative stress block ciliary neurotrophic factor activation of Jak/STAT signaling in neurons. J Neurochem.

[217] Planas AM, Gorina R, Chamorro A (2006). Signalling pathways mediating inflammatory responses in brain ischaemia. Biochem Soc Trans.

[218] Kacimi R, Giffard RG, Yenari MA (2011). Endotoxin-activated microglia injure brain derived endothelial cells via NF-κB, JAK-STAT and JNK stress kinase pathways. J Inflamm (Lond).

[219] Chen Y, Qin C, Huang J, Tang X, Liu C, Huang K (2020). The role of astrocytes in oxidative stress of central nervous system: A mixed blessing. Cell Prolif.

[220] Wanner IB, Anderson MA, Song B, Levine J, Fernandez A, Gray-Thompson Z (2013). Glial scar borders are formed by newly proliferated, elongated astrocytes that interact to corral inflammatory and fibrotic cells via STAT3-dependent mechanisms after spinal cord injury. J Neurosci.

[221] Lian H, Yang L, Cole A, Sun L, Chiang AC-A, Fowler SW (2015). NFκB-activated astroglial release of complement C3 compromises neuronal morphology and function associated with Alzheimer’s disease. Neuron..

[222] Lee KH, Cha M, Lee BH (2021). Crosstalk between Neuron and Glial Cells in Oxidative Injury and Neuroprotection. Int J Mol Sci.

[223] Edelmann E, Lessmann V, Brigadski T (2014). Pre- and postsynaptic twists in BDNF secretion and action in synaptic plasticity. Neuropharmacology..

[224] Lima Giacobbo B, Doorduin J, Klein HC, Dierckx RAJO, Bromberg E, de Vries EFJ (2019). Brain-Derived Neurotrophic Factor in Brain Disorders: Focus on Neuroinflammation. Mol Neurobiol.

[225] Porter GA, O’Connor JC (2022). Brain-derived neurotrophic factor and inflammation in depression: Pathogenic partners in crime?. World J Psychiatry.

[226] Kaplan DR, Miller FD (1997). Signal transduction by the neurotrophin receptors. Curr Opin Cell Biol.

[227] Lund IV, Hu Y, Raol YH, Benham RS, Faris R, Russek SJ (2008). BDNF selectively regulates GABAA receptor transcription by activation of the JAK/STAT pathway. Sci Signal..

[228] Hixson KM, Cogswell M, Brooks-Kayal AR, Russek SJ (2019). Evidence for a non-canonical JAK/STAT signaling pathway in the synthesis of the brain’s major ion channels and neurotransmitter receptors. BMC Genomics.

[229] Lee C-H, Giuliani F (2019). The Role of Inflammation in Depression and Fatigue. Front Immunol.

[230] Zhang Z-Q, Wang X, Xue B-H, Zhao Y, Xie F, Wang S-D (2021). Chronic stress promotes glioma cell proliferation via the PI3K/Akt signaling pathway. Oncol Rep.

[231] Zhou Z, Chen H, Tang X, He B, Gu L, Feng H (2022). Total Saikosaponins Attenuates Depression-Like Behaviors Induced by Chronic Unpredictable Mild Stress in Rats by Regulating the PI3K/AKT/NF-κB Signaling Axis. Evid Based Complement Alternat Med: eCAM.

[232] Peterson WM, Wang Q, Tzekova R, Wiegand SJ (2000). Ciliary neurotrophic factor and stress stimuli activate the JAK-STAT pathway in retinal neurons and glia. J Neurosci.

[233] Byrne CJ, Khurana S, Kumar A, Tai TC (2018). Inflammatory Signaling in Hypertension: Regulation of Adrenal Catecholamine Biosynthesis. Front Endocrinol.

[234] Bunn SJ, Ait-Ali D, Eiden LE (2012). Immune-neuroendocrine integration at the adrenal gland: cytokine control of the adrenomedullary transcriptome. J Mol Neurosci.

[235] Nater UM, Whistler T, Lonergan W, Mletzko T, Vernon SD, Heim C (2009). Impact of acute psychosocial stress on peripheral blood gene expression pathways in healthy men. Biol Psychol.

[236] Dong J, Li J, Cui L, Wang Y, Lin J, Qu Y, Wang H (2018). Cortisol modulates inflammatory responses in LPS-stimulated RAW264.7 cells via the NF-κB and MAPK pathways. BMC Vet Res..

[237] Castillo J, Teles M, Mackenzie S, Tort L (2009). Stress-related hormones modulate cytokine expression in the head kidney of gilthead seabream (Sparus aurata). Fish Shellfish Immunol.

[238] Swain P, Nayak SK, Nanda PK, Dash S (2008). Biological effects of bacterial lipopolysaccharide (endotoxin) in fish: a review. Fish Shellfish Immunol.

[239] Kimura A, Naka T, Muta T, Takeuchi O, Akira S, Kawase I (2005). Suppressor of cytokine signaling-1 selectively inhibits LPS-induced IL-6 production by regulating JAK-STAT. Proc Natl Acad Sci USA.

[240] Yeager MP, Pioli PA, Guyre PM (2011). Cortisol exerts bi-phasic regulation of inflammation in humans. Dose Response.

[241] Croker BA, Kiu H, Nicholson SE (2008). SOCS regulation of the JAK/STAT signalling pathway. Semin Cell Dev Biol.

[242] Philip AM, Jørgensen EH, Maule AG, Vijayan MM (2014). Tissue-specific molecular immune response to lipopolysaccharide challenge in emaciated anadromous Arctic charr. Dev Comp Immunol.

[243] Philip AM, Vijayan MM (2015). Stress-Immune-Growth Interactions: Cortisol Modulates Suppressors of Cytokine Signaling and JAK/STAT Pathway in Rainbow Trout Liver. PLoS ONE.

[244] Schafroth U, Godang K, Ueland T, Bollerslev J (2001). Leptin response to endogenous acute stress is independent of pituitary function. Eur J Endocrinol.

[245] Kain ZN, Zimolo Z, Heninger G (1999). Leptin and the perioperative neuroendocrinological stress response. J Clin Endocrinol Metab.

[246] Triantafyllou GA, Paschou SA, Mantzoros CS (2016). Leptin and Hormones: Energy Homeostasis. Endocrinol Metab Clin North Am.

[247] Park H-K, Ahima RS (2015). Physiology of leptin: energy homeostasis, neuroendocrine function and metabolism. Metabolism..

[248] Laferrère B, Fried SK, Osborne T, Pi-Sunyer FX (2000). Effect of one morning meal and a bolus of dexamethasone on 24-hour variation of serum leptin levels in humans. Obes Res.

[249] Bouillon-Minois J-B, Trousselard M, Thivel D, Benson AC, Schmidt J, Moustafa F, Bouvier D, Dutheil F (2021). Leptin as a Biomarker of Stress: A Systematic Review and Meta-Analysis. Nutrients.

[250] Lu X-Y, Kim CS, Frazer A, Zhang W (2006). Leptin: a potential novel antidepressant. Proc Natl Acad Sci USA.

[251] Lei Y, Wang D, Bai Y, Nougaisse J, Weintraub NL, Guo M (2022). Leptin enhances social motivation and reverses chronic unpredictable stress-induced social anhedonia during adolescence. Mol Psychiatry.

[252] Fernández-Riejos P, Najib S, Santos-Alvarez J, Martín-Romero C, Pérez-Pérez A, González-Yanes C (2010). Role of leptin in the activation of immune cells. Mediators Inflamm.

[253] La Cava A, Matarese G (2004). The weight of leptin in immunity. Nat Rev Immunol.

[254] Reindl KM, Kittilson JD, Bergan HE, Sheridan MA (2011). Growth hormone-stimulated insulin-like growth factor-1 expression in rainbow trout (Oncorhynchus mykiss) hepatocytes is mediated by ERK, PI3K-AKT, and JAK-STAT. Am J Physiol Regul Integr Comp Physiol.

[255] Wang X, Jiang J, Warram J, Baumann G, Gan Y, Menon RK (2008). Endotoxin-induced proteolytic reduction in hepatic growth hormone (GH) receptor: a novel mechanism for GH insensitivity. Mol Endocrinol.

[256] Natarajan R, Forrester L, Chiaia NL, Yamamoto BK (2017). Chronic-Stress-Induced Behavioral Changes Associated with Subregion-Selective Serotonin Cell Death in the Dorsal Raphe. J Neurosci.

[257] Vahid-Ansari F, Albert PR (2021). Rewiring of the Serotonin System in Major Depression. Front Psych.

[258] Kong E, Sucic S, Monje FJ, Savalli G, Diao W, Khan D (2015). STAT3 controls IL6-dependent regulation of serotonin transporter function and depression-like behavior. Sci Rep.

[259] Gulbins A, Grassmé H, Hoehn R, Kohnen M, Edwards MJ, Kornhuber J (2016). Role of Janus-Kinases in Major Depressive Disorder. Neurosignals.

[260] Benkortbi Elouaer AAE, Ben Mohamed B, Zaafrane F, Gaha L, Bel Hadj Jrad Tensaout B (2023). Case control study: G-allele of rs4244165 in JAK1 gene correlated with high-level brief psychiatric rating scale in bipolar patients. Medicine (Baltimore)..

[261] Long Y, Wang Y, Shen Y, Huang J, Li Y, Wu R (2023). Minocycline and antipsychotics inhibit inflammatory responses in BV-2 microglia activated by LPS via regulating the MAPKs/ JAK-STAT signaling pathway. BMC Psychiatry.

[262] Almutabagani LF, Almanqour RA, Alsabhan JF, Alhossan AM, Alamin MA, Alrajeh HM (2023). Inflammation and Treatment-Resistant Depression from Clinical to Animal Study: A Possible Link?. Neurol Int.

[263] Lee HC, Tan KL, Cheah PS, Ling KH (2016). Potential Role of JAK-STAT Signaling Pathway in the Neurogenic-to-Gliogenic Shift in Down Syndrome Brain. Neural Plast.

[264] Borbély É, Simon M, Fuchs E, Wiborg O, Czéh B, Helyes Z (2022). Novel drug developmental strategies for treatment-resistant depression. Br J Pharmacol.

[265] Shariq AS, Brietzke E, Rosenblat JD, Pan Z, Rong C, Ragguett RM (2018). Therapeutic potential of JAK/STAT pathway modulation in mood disorders. Rev Neurosci.

[266] Gałecka M, Szemraj J, Su K-P, Halaris A, Maes M, Skiba A (2022). Is the JAK-STAT Signaling Pathway Involved in the Pathogenesis of Depression?. J Clin Med.

[267] Dai X-Y, Liu L, Song F-H, Gao S-J, Wu J-Y, Li D-Y, et al. Targeting the JAK2/STAT3 signaling pathway for chronic pain. Aging Dis. 2023. 10.14336/AD.2023.0515. 10.14336/AD.2023.0515 PMC10796104 37307838

[268] Yan D, Fan H, Chen M, Xia L, Wang S, Dong W (2022). The efficacy and safety of JAK inhibitors for alopecia areata: A systematic review and meta-analysis of prospective studies. Front Pharmacol.

[269] Shawky AM, Almalki FA, Abdalla AN, Abdelazeem AH, Gouda AM (2022). A Comprehensive Overview of Globally Approved JAK Inhibitors. Pharmaceutics.

[270] Gupta N, Papasotiriou S, Hanauer S (2023). The evolving role of JAK inhibitors in the treatment of inflammatory bowel disease. Expert Rev Clin Immunol.

[271] Wu C-Y, Yang H-Y, Lai J-H (2023). Potential therapeutic targets beyond cytokines and Janus kinases for autoimmune arthritis. Biochem Pharmacol.

[272] Shamail GMH, Haridoss M, Natarajan M, Joshua V, Bagepally BS (2022). Association Between Janus Kinase Inhibitors Therapy and Mental Health Outcome in Rheumatoid Arthritis: A Systematic Review and Meta-analysis. Rheumatol Ther.

[273] Genovese MC, Fleischmann R, Combe B, Hall S, Rubbert-Roth A, Zhang Y (2018). Safety and efficacy of upadacitinib in patients with active rheumatoid arthritis refractory to biologic disease-modifying anti-rheumatic drugs (SELECT-BEYOND): a double-blind, randomised controlled phase 3 trial. Lancet.

[274] Combe B, Kivitz A, Tanaka Y, der Heijde DV, Simon-Campos JA, Baraf HSB (2020). Thu0198 Efficacy and Safety of Filgotinib for Patients with Rheumatoid Arthritis with Inadequate Response to Methotrexate: Finch 1 52-Week Results. Ann Rheum Dis.

[275] Tanaka Y, Matsubara T, Atsumi T, Amano K, Ishiguro N, Sugiyama E (2022). Efficacy and safety of filgotinib in combination with methotrexate in Japanese patients with active rheumatoid arthritis who have an inadequate response to methotrexate: Subpopulation analyses of 24-week data of a global phase 3 study (FINCH 1). Mod Rheumatol.

[276] Burmester GR, Rigby WF, van Vollenhoven RF, Kay J, Rubbert-Roth A, Kelman A (2016). Tocilizumab in early progressive rheumatoid arthritis: FUNCTION, a randomised controlled trial. Ann Rheum Dis.

[277] Kavanagh ME, Horning BD, Khattri R, Roy N, Lu JP, Whitby LR (2022). Selective inhibitors of JAK1 targeting an isoform-restricted allosteric cysteine. Nat Chem Biol.

[278] Virtanen A, Palmroth M, Liukkonen S, Kurttila A, Haikarainen T, Isomäki P (2023). Differences in JAK Isoform Selectivity Among Different Types of JAK Inhibitors Evaluated for Rheumatic Diseases Through In Vitro Profiling. Arthritis Rheumatol.

[279] Torchilin VP (2014). Multifunctional, stimuli-sensitive nanoparticulate systems for drug delivery. Nat Rev Drug Discov.

[280] Smith P, Yao W, Shepard S, Covington M, Lee J, Lofland J (2021). Developing a JAK Inhibitor for Targeted Local Delivery: Ruxolitinib Cream. Pharmaceutics.

[281] Taylor PC, Laedermann C, Alten R, Feist E, Choy E, Haladyj E (2023). A JAK Inhibitor for Treatment of Rheumatoid Arthritis: The Baricitinib Experience. J Clin Med.

[282] Slamon DJ, Leyland-Jones B, Shak S, Fuchs H, Paton V, Bajamonde A (2001). Use of chemotherapy plus a monoclonal antibody against HER2 for metastatic breast cancer that overexpresses HER2. N Engl J Med.

[283] Herrera-deGuise C, Serra-Ruiz X, Lastiri E, Borruel N (2023). JAK inhibitors: A new dawn for oral therapies in inflammatory bowel diseases. Front Med (Lausanne).

[284] Riggs LM, Gould TD (2021). Ketamine and the Future of Rapid-Acting Antidepressants. Annu Rev Clin Psychol.

[285] Lofts A, Abu-Hijleh F, Rigg N, Mishra RK, Hoare T (2022). Using the Intranasal Route to Administer Drugs to Treat Neurological and Psychiatric Illnesses: Rationale, Successes, and Future Needs. CNS Drugs.

[286] Islam SU, Shehzad A, Ahmed MB, Lee YS (2020). Intranasal Delivery of Nanoformulations: A Potential Way of Treatment for Neurological Disorders. Molecules.

[287] Formica ML, Real DA, Picchio ML, Catlin E, Donnelly RF, Paredes AJ (2022). On a highway to the brain: A review on nose-to-brain drug delivery using nanoparticles. Appl Mater Today.

[288] Liu X (2011). Clinical trials of intranasal delivery for treating neurological disorders–a critical review. Expert Opin Drug Deliv.

[289] Lukas C, Bellenberg B, Hahn HK, Rexilius J, Drescher R, Hellwig K (2009). Benefit of repetitive intrathecal triamcinolone acetonide therapy in predominantly spinal multiple sclerosis: prediction by upper spinal cord atrophy. Ther Adv Neurol Disord.

[290] Montero P, Milara J, Roger I, Cortijo J (2021). Role of JAK/STAT in Interstitial Lung Diseases; Molecular and Cellular Mechanisms. Int J Mol Sci.

[291] Gajjela BK, Zhou MM (2022). Calming the cytokine storm of COVID-19 through inhibition of JAK2/STAT3 signaling. Drug Discov Today.

[292] Gusev E, Sarapultsev A, Solomatina L, Chereshnev V (2022). SARS-CoV-2-Specific Immune Response and the Pathogenesis of COVID-19. Int J Mol Sci.

[293] Jain NK, Tailang M, Jain HK, Chandrasekaran B, Sahoo BM, Subramanian A (2023). Therapeutic implications of current Janus kinase inhibitors as anti-COVID agents: A review. Front Pharmacol.

[294] Tanaka Y, Takeuchi T, Yamanaka H, Nakamura H, Toyoizumi S, Zwillich S (2015). Efficacy and safety of tofacitinib as monotherapy in Japanese patients with active rheumatoid arthritis: a 12-week, randomized, phase 2 study. Mod Rheumatol.

[295] Patel P, Patel S, Chudasama P, Soni S, Raval M (2023). Roflumilast ameliorates diabetic nephropathy in rats through down-regulation of JAK/STAT signaling pathway. Naunyn Schmiedebergs Arch Pharmacol.

[296] Baghdassarian H, Blackstone SA, Clay OS, Philips R, Matthiasardottir B, Nehrebecky M (2023). Variant STAT4 and Response to Ruxolitinib in an Autoinflammatory Syndrome. N Engl J Med.

[297] Bharadwaj U, Kasembeli MM, Robinson P, Tweardy DJ (2020). Targeting Janus Kinases and Signal Transducer and Activator of Transcription 3 to Treat Inflammation, Fibrosis, and Cancer: Rationale, Progress, and Caution. Pharmacol Rev.

[298] Liongue C, Ward AC (2013). Evolution of the JAK-STAT pathway. JAK-STAT.

[299] Qu L, Matz AJ, Karlinsey K, Cao Z, Vella AT, Zhou B (2022). Macrophages at the Crossroad of Meta-Inflammation and Inflammaging. Genes (Basel).

[300] Cevenini E, Monti D, Franceschi C (2013). Inflamm-ageing. Curr Opin Clin Nutr Metab Care.

[301] Songkiatisak P, Rahman SMT, Aqdas M, Sung M-H (2022). NF-κB, a culprit of both inflamm-ageing and declining immunity?. Immun Ageing.

[302] Wu S, Wolfe A (2012). Signaling of cytokines is important in regulation of GnRH neurons. Mol Neurobiol.

[303] de Oliveira CMB, Sakata RK, Issy AM, Gerola LR, Salomão R. Cytokines and pain. Rev Bras Anestesiol. 2011;61(2):255–59, 260–265, 137–142. 10.1016/S0034-7094(11)70029-0. 10.1016/S0034-7094(11)70029-0 21474032

[304] Johnston EK, Abbott RD (2023). Adipose Tissue Paracrine-, Autocrine-, and Matrix-Dependent Signaling during the Development and Progression of Obesity. Cells.

[305] Huang PL (2009). A comprehensive definition for metabolic syndrome. Dis Model Mech.

[306] Furuta K, Tang X, Islam S, Tapia A, Chen ZB, Ibrahim SH (2023). Endotheliopathy in the metabolic syndrome: Mechanisms and clinical implications. Pharmacol Ther.

[307] Gurzov EN, Stanley WJ, Pappas EG, Thomas HE, Gough DJ (2016). The JAK/STAT pathway in obesity and diabetes. FEBS J.

[308] Hue L, Taegtmeyer H (2009). The Randle cycle revisited: a new head for an old hat. Am J Physiol Endocrinol Metab.

[309] Labadie P (1976). Glucose-alanine cycle. Rev Prat.

[310] Graham E, Deschênes SS, Rosella LC, Schmitz N (2021). Measures of depression and incident type 2 diabetes in a community sample. Ann Epidemiol.

[311] Ambrósio G, Kaufmann FN, Manosso L, Platt N, Ghisleni G, Rodrigues ALS (2018). Depression and peripheral inflammatory profile of patients with obesity. Psychoneuroendocrinology.

[312] Gusev E, Solomatina L, Zhuravleva Y, Sarapultsev A (2021). The Pathogenesis of End-Stage Renal Disease from the Standpoint of the Theory of General Pathological Processes of Inflammation. Int J Mol Sci.

[313] Thakur S, Dhapola R, Sarma P, Medhi B, Reddy DH (2023). Neuroinflammation in Alzheimer’s Disease: Current Progress in Molecular Signaling and Therapeutics. Inflammation.

[314] Isik S, Yeman Kiyak B, Akbayir R, Seyhali R, Arpaci T (2023). Microglia Mediated Neuroinflammation in Parkinson’s Disease. Cells.

[315] Miller AH, Maletic V, Raison CL (2009). Inflammation and its discontents: the role of cytokines in the pathophysiology of major depression. Biol Psychiatry.

[316] Varesi A, Campagnoli LIM, Chirumbolo S, Candiano B, Carrara A, Ricevuti G (2023). The brain-gut-microbiota interplay in depression: A key to design innovative therapeutic approaches. Pharmacol Res.

[317] Zhang H, Wang Z, Wang G, Song X, Qian Y, Liao Z (2023). Understanding the Connection between Gut Homeostasis and Psychological Stress. J Nutr.

[318] Shamabadi A, Akhondzadeh S (2023). Inflammation-Schizophrenia: A Bidirectional Causal Association Mediated by Cytokines. Avicenna J Med Biotechnol..

[319] Goldsmith DR, Bekhbat M, Mehta ND, Felger JC (2023). Inflammation-Related Functional and Structural Dysconnectivity as a Pathway to Psychopathology. Biol Psychiatry.

[320] Johnson DE, O’Keefe RA, Grandis JR (2018). Targeting the IL-6/JAK/STAT3 signalling axis in cancer. Nat Rev Clin Oncol.

[321] Knottnerus SJG, Bleeker JC, Wüst RCI, Ferdinandusse S, Wijburg FA, IJlst L (2018). Disorders of mitochondrial long-chain fatty acid oxidation and the carnitine shuttle. Rev Endocr Metab Disord..

[322] Nagappan PG, Chen H, Wang D-Y (2020). Neuroregeneration and plasticity: a review of the physiological mechanisms for achieving functional recovery postinjury. Mil Med Res.

[323] Mattson MP, Magnus T (2006). Ageing and neuronal vulnerability. Nat Rev Neurosci.

[324] Bourgognon J-M, Cavanagh J (2020). The role of cytokines in modulating learning and memory and brain plasticity. Brain Neurosci Adv..

[325] McGregor G, Irving AJ, Harvey J (2017). Canonical JAK-STAT signaling is pivotal for long-term depression at adult hippocampal temporoammonic-CA1 synapses. FASEB J.

