# Supplementary material for: JAK-STAT signaling in inflammation and stress-related diseases: implications for therapeutic interventions
Source: Mol Biomed. 2023 Nov 8;4:40. doi: 10.1186/s43556-023-00151-1 (PMC10632324; doi:10.1186/s43556-023-00151-1)
Supplement: Supplementary file 1 — Additional file 1: Supplementary Table S1. List of JAK inhibitor’s studies, registered in Clinical Trials Registry (June 2023). [file 43556_2023_151_MOESM1_ESM.docx]

**JAK-STAT Signaling in Inflammation and Stress-Related Diseases: Implications for Therapeutic Interventions**

Alexey Sarapultsev^1,2*^, Evgenii Gusev^1,2#^, Maria Komelkova^1,2^, Irina Utepova^2,3^, Shanshan Luo^4^, Desheng Hu^5,6,7#^

^1^ Russian-Chinese Education and Research Center of System Pathology, South Ural State University, 454080 Chelyabinsk, Russia

^2^ Institute of Immunology and Physiology, Ural Branch of the Russian Academy of Science, 620049 Ekaterinburg, Russia

^3^ Department of Organic and Biomolecular Chemistry, Ural Federal University, 28 Mira str., 620002, Ekaterinburg, Russian Federation

^4^ Institute of Hematology, Union Hospital, Tongji Medical College, Huazhong University of Science and Technology, Wuhan, 430022 China

^5^ Department of Integrated Traditional Chinese and Western Medicine, Union Hospital, Tongji Medical College, Huazhong University of Science and Technology, 430022 Wuhan, China

^6^ Key Laboratory of Biological Targeted Therapy, The Ministry of Education, 430022 Wuhan, China

^7^ Clinical Research Center of Cancer Immunotherapy, 430022 Hubei Wuhan, China

*Correspondence:

Alexey Sarapultsev

a.sarapultsev@gmail.com

Russian-Chinese Education and Research Center of System Pathology, South Ural State University, 454080 Chelyabinsk, Russia

**Supplementary Table S1**. List of JAK inhibitor’s studies, registered in Clinical Trials Registry (June 2023).

| **NCT Number** | **Conditions** | **Study Design** | **Interventions** | **JAK1 inhibitor’s type** | **Study Type** | **Phase** | **Study Start** | **Study Completion** |
| --- | --- | --- | --- | --- | --- | --- | --- | --- |
| Active, not recruiting | | | | | | | | |
| NCT05090410 | Rheumatoid Arthritis | Primary Purpose: Treatment  Allocation: Randomized | Filgotinib 200mg/day  subcutaneous tocilizumab 162mg/biweekly | selective JAK1 inhibitor | INT | 3 | 03.03.2021 | 31.12.2023 |
| NCT05069831 | Food Allergy | Primary Purpose: Treatment  Allocation: Randomized | Abrocitinib | selective JAK1 inhibitor | INT | 1 | 16.05.2022 | 2024-10 |
| NCT05121298 | Rheumatoid Arthritis | Primary Purpose: Treatment  Allocation: Non-Randomized | Upadacitinib 15mg/day | selective JAK1 inhibitor | INT | 3 | 12.01.2021 | 30.09.2024 |
| NCT04530305 | Rheumatoid Arthritis | Observational Model: Other  Time Perspective: Prospective | Drug: Upadacitinib <15 MG [Rinvoq] | selective JAK1 inhibitor | OBS |  | 07.01.2021 | 2023-10 |
| NCT05456412 | Colitis, Ulcerative | Primary Purpose: Basic Science | JAK inhibitor treatment | NA | INT | NA | 20.12.2022 | 2026-09 |
| NCT05451615 | Abatacept  Treatment Compliance  Rheumatoid Arthritis | Primary Purpose: Treatment  Allocation: Randomized | Janus Kinase Inhibitor  Abatacept | NA | INT | 3 | 30.09.2022 | 30.06.2024 |
| NCT05393674 | Primary and Secondary Myelofibrosis | Primary Purpose: Treatment  Allocation: N/A | Fedratinib Oral Capsule [Inrebic]  Nivolumab | semi-selective JAK-2 inhibitor | INT | 2 | 14.06.2022 | 30.06.2026 |
| NCT05177471 | Systemic Sclerosis  Interstitial Lung Disease | Observational Model: Cohort  Time Perspective: Retrospective |  | NA | OBS |  | 19.01.2022 | 04.07.2024 |
| NCT04602091 | Rheumatoid Arthritis  Inflammatory Rheumatism  Psoriatic Arthritis | Observational Model: Cohort  Time Perspective: Prospective | Other: no intervention | NA | OBS |  | 08.10.2019 | 2029-01 |
| NCT04576156 | Myelofibrosis | Primary Purpose: Treatment  Allocation: Randomized | Imetelstat  Best Available Therapy (BAT) | NA | INT | 3 | 12.04.2021 | 27.04.2026 |
| NCT04370301 | Primary and Secondary Myelofibrosis | Primary Purpose: Treatment  Allocation: N/A | Cyclophosphamide  JAK Inhibitor  Fludarabine | NA | INT | 2 | 09.02.2021 | 31.08.2029 |
| NCT03662126 | Primary Myelofibrosis  Post-Polycythemia Vera MF  Post-Essential Thrombocythemia MF | Primary Purpose: Treatment  Allocation: Randomized | KRT-232  Drug: Best Available Therapy (BAT) | NA | INT | 2-3 | 15.01.2019 | 31.12.2025 |
| NCT04239989 | Bronchiolitis Obliterans | Primary Purpose: Treatment  Allocation: N/A | Itacitinib  Itacitinib Adipate | selective JAK1 inhibitor | INT | 1 | 08.04.2021 | 31.05.2024 |
| NCT05279001 | Myelofibrosis | Primary Purpose: Treatment  Allocation: N/A | Jaktinib Hydrochloride Tablet | JAK 1, JAK 2, JAK 3 and AVCR1 inhibitor | INT | 1 | 01.07.2022 | 30.11.2023 |
| NCT04246372 | Down Syndrome  Alopecia Areata  Atopic Dermatitis / Eczema | Primary Purpose: Treatment  Allocation: N/A | Drug: Tofacitinib | JAK1, JAK3 inhibitor | INT | 2 | 21.10.2020 | 2024-12 |
| NCT05387980 | Atopic Dermatitis | Observational Model: Cohort  Time Perspective: Prospective | Drug: CIBINQO (abrocitinib) | selective JAK1 inhibitor | OBS |  | 19.04.2022 | 30.06.2027 |
| NCT04640532 | Myelofibrosis  Post-PV MF  Post-ET Myelofibrosis | Primary Purpose: Treatment  Allocation: Randomized | Drug: KRT-232  Drug: TL-895 | NA | INT | 1-2 | 17.11.2020 | 24.07.2025 |
| NCT05250115 | Dermatitis, Atopic | Observational Model: Cohort  Time Perspective: Prospective | CIBINQO (abrocitinib) | selective JAK1 inhibitor | OBS |  | 10.05.2022 | 14.04.2025 |
| NCT05374785 | Rheumatoid Arthritis | Primary Purpose: Treatment  Allocation: Randomized | Drug: CPL409116  Other: Placebo | JAK1 and JAK3 inhibitor with lower inhibitory activity against JAK2 and Tyk2. | INT | 2 | 01.05.2022 | 31.08.2023 |
| NCT05906628 | Hand Eczema | Primary Purpose: Treatment  Allocation: Randomized | Drug: Ruxolitinib cream  Drug: Vehicle | JAK1 and JAK2 inhibitor | INT | 2 | 14.08.2023 | 17.01.2025 |
| NCT05016297 | Sjogren's Syndrome | Primary Purpose: Treatment  Allocation: Randomized | Drug: Baricitinib  Drug: Hydroxychloroquine | JAK1 and JAK2 inhibitor | INT | 2 | 14.07.2022 | 15.10.2024 |
| NCT04717414 | Myeloproliferative Disorders  Myelofibrosis  Primary Myelofibrosis | Primary Purpose: Treatment  Allocation: Randomized | Drug: ACE-536  Other: Placebo | NA (JAK2 inhibitor) | INT | 3 | 25.02.2021 | 23.08.2025 |
| NCT04282187 | Acute Myeloid Leukemia  Essential Thrombocythemia  Myelodysplastic Syndrome | Primary Purpose: Treatment  Allocation: N/A | Drug: Decitabine  Drug: Ruxolitinib  Drug: Fedratinib | JAK1 and JAK2 inhibitor (Ruxolitinib)  JAK2 inhibitor (Fedratinib) | INT | 2 | 24.03.2020 | 11.11.2024 |
| NCT05313620 | Ulcerative Colitis  Thromboembolism | Primary Purpose: Treatment  Allocation: Non-Randomized | Drug: Tofacitinib  Drug: Infliximab, Adalimumab, Golimumab | JAK1, JAK3 inhibitor (Tofacitinib) | INT | 4 | 01.04.2022 | 2024-10 |
| NCT03755414 | Acute Myelogenous Leukemia  Acute Lymphocytic Leukemia  Myelodysplastic Syndromes | Primary Purpose: Treatment  Allocation: Non-Randomized | Procedure: Stem cell transplantation  Drug: Itacitinib  Other: Functional Assessment of Cancer Therapy-Bone Marrow Transplant | selective JAK1 inhibitor | INT | 1 | 04.09.2019 | 13.05.2024 |
| NCT05689151 | Atopic Dermatitis  Eczema | Observational Model: Cohort  Time Perspective: Prospective | Abrocitinib | selective JAK1 inhibitor | OBS |  | 09.03.2023 | 31.05.2027 |
| NCT04496960 | Sjogren's Syndrome | Primary Purpose: Treatment  Allocation: Randomized | Drug: tofacitinib  Other: Placebo | JAK1, JAK3 inhibitor (Tofacitinib) | INT | 1-2 | 18.05.2021 | 22.09.2025 |
| NCT04821206 | Arthritis, Rheumatoid  Arthritis, Psoriatic  Spondyloarthritis | Observational Model: Cohort  Time Perspective: Prospective | Drug: DMARDs, biologic DMARDs  JAK Inhibitor | NA | OBS |  | 01.03.2021 | 01.07.2024 |
| NCT04655118 | Myelofibrosis | Primary Purpose: Treatment  Allocation: Randomized | Drug: TL-895 | NA | INT | 2 | 22.10.2020 | 01.12.2025 |
| NCT04105010 | Relapsed or Refractory Peripheral T Cell Lymphoma | Primary Purpose: Treatment  Allocation: Non-Randomized | Drug: AZD4205 (Golidocitinib) | selective JAK1 inhibitor | INT | 2 | 10.09.2019 | 2024-12 |
| NCT05696795 | Sarcoidosis | Primary Purpose: Treatment | Drug: Abrocitinib 200 mg | selective JAK1 inhibitor | INT | 2 | 01.06.2023 | 01.03.2024 |
| NCT05198310 | Arthritis, Rheumatoid | Primary Purpose: Treatment  Allocation: Randomized | Drug: KPL-404  Drug: Placebo | NA | INT | 2 | 14.12.2021 | 2024-03 |
| NCT04562389 | Myelofibrosis | Primary Purpose: Treatment  Allocation: Randomized | Drug: Selinexor | NA | INT | 3 | 11.03.2021 | 2028-03 |
| NCT03976245 | Rheumatoid Arthritis | Primary Purpose: Treatment  Allocation: Randomized | Drug: Etanercept  Drug: tofacitinib | JAK1, JAK3 inhibitor (Tofacitinib) | INT | 4 | 01.03.2020 | 2023-03 |
| NCT03549416 | Atopic Dermatitis | Observational Model: Other |  | NA | OBS |  | 01.01.2018 | 31.12.2028 |
| Active, not recruiting | | | | | | | | |
| NCT05247489 | Vitiligo  JAK Inhibitor | Primary Purpose: Treatment  Allocation: Randomized | Drug: Ruxolitinib 1.5% cream  Device: NB-UVB phototherapy | JAK1 and JAK2 inhibitor | INT | 2 | 05.05.2022 | 23.01.2024 |
| NCT05120362 | Acneiform Eruptions | Primary Purpose: Treatment | Drug: Cream containing JAK Inhibitor | NA | INT | NA | 03.11.2021 | 30.04.2023 |
| NCT03921554 | Aicardi Goutieres Syndrome | Primary Purpose: Treatment  Allocation: N/A | Drug: Baricitinib | JAK1 and JAK2 inhibitor | INT | 2 | 03.06.2019 | 2025-12 |
| NCT04208464 | Idiopathic Inflammatory Myopathies | Primary Purpose: Treatment  Allocation: Randomized | Drug: Baricitinib | JAK1 and JAK2 inhibitor | INT | 2 | 07.10.2021 | 25.09.2023 |
| NCT02251821 | Primary Myelofibrosis  Secondary Myelofibrosis | Primary Purpose: Treatment  Allocation: N/A | Procedure: Allogeneic Hematopoietic Stem Cell Transplantation  Drug: Busulfan  Drug: Cyclophosphamide | JAK1 and JAK2 inhibitor (Ruxolitinib) | INT | 2 | 20.10.2014 | 28.12.2025 |
| NCT05767775 | Rheumatoid Arthritis | Observational Model: Case-Control  Time Perspective: Prospective | Other: Assessment of synovial- and adipose tissue-derived inflammatory biomarkers | NA | OBS |  | 01.06.2019 | 15.03.2023 |
| NCT02723994 | Leukemia | Primary Purpose: Treatment  Allocation: N/A | Drug: Ruxolitinib  Drug: Asparaginase Erwinia Chrysanthemi  Drug: Cyclophosphamide  10 more | JAK1 and JAK2 inhibitor | INT | 2 | 30.09.2016 | 28.11.2025 |
| NCT05638932 | COVID-19 | Observational Model: Cohort  Time Perspective: Retrospective | Drug: Initiation of IL6Ri (tocilizumab or sarilumab) versus JAKi (baricitinib or tofacitinib) added to systemic corticosteroids of interest (CSI) | JAK1 and JAK2 inhibitor | OBS |  | 16.06.2020 | 2023-09 |
| NCT04173494 | Primary Myelofibrosis  Post-polycythemia Vera Myelofibrosis  Post-essential Thrombocythemia Myelofibrosis | Primary Purpose: Treatment  Allocation: Randomized | Drug: Momelotinib  Drug: Danazol  Drug: Placebo to match momelotinib  1 more | JAK1 and JAK2 inhibitor | INT | 3 | 2020-02-07 | 2028-04 |
| NCT05456529 | Atopic Dermatitis (AD) | Primary Purpose: Treatment | Drug: Ruxolitinib Cream | JAK1 and JAK2 inhibitor | INT | 3 | 01.09.2022 | 29.04.2024 |
| NCT04006457 | Alopecia Areata | Primary Purpose: Treatment  Allocation: Non-Randomized | Drug: PF-06651600  Biological: Tetanus and diphtheria toxoids and acellular pertussis (Tdap) vaccine  Biological: Meningococcal (groups A, C, W-135 and Y [ACWY]) oligosaccharide diphtheria CRM197 conjugate vaccine | JAK3/TEC inhibitor (PF-06651600) | INT | 3 | 18.07.2019 | 03.02.2026 |
| NCT04206644 | Systemic Sclerosis | Observational Model: Other  Time Perspective: Cross-Sectional | Other: biological analysis | JAK1 and JAK2 inhibitor (Ruxolitinib) | OBS |  | 21.01.2021 | 13.02.2027 |
| NCT03954236 | Non-sclerotic Cutaneous Chronic Graft-versus-host Disease | Primary Purpose: Treatment  Allocation: Randomized | Drug: topical ruxolitinib 1.5% cream  Other: Topical vehicle/moisturizer cream | JAK1 and JAK2 inhibitor | INT | 2 | 14.05.2019 | 2024-05 |
| NCT03584516 | Chronic Graft-versus-host Disease | Primary Purpose: Treatment  Allocation: Randomized | Drug: Itacitinib  Drug: Placebo  Drug: Methylprednisolone | Selective JAK1 inhibitor | INT | 2 | 17.01.2019 | 08.09.2023 |
| NCT04071366 | Cytokine Release Syndrome | Primary Purpose: Prevention  Allocation: Randomized | Drug: Itacitinib  Drug: Immune effector cell therapy  Drug: Placebo | Selective JAK1 inhibitor | INT | 2 | 07.02.2020 | 22.08.2023 |
